# Finite-Size Effects on Traveling Wave Solutions to Neural Field Equations

**DOI:** 10.1186/s13408-017-0048-2

**Published:** 2017-07-06

**Authors:** Eva Lang, Wilhelm Stannat

**Affiliations:** 1 Institut für Mathematik, Technische Universität Berlin, Berlin, 10623 Germany; 2 Bernstein Center for Computational Neuroscience, Berlin, 10115 Germany

## Abstract

Neural field equations are used to describe the spatio-temporal evolution of the activity in a network of synaptically coupled populations of neurons in the continuum limit. Their heuristic derivation involves two approximation steps. Under the assumption that each population in the network is large, the activity is described in terms of a population average. The discrete network is then approximated by a continuum. In this article we make the two approximation steps explicit. Extending a model by Bressloff and Newby, we describe the evolution of the activity in a discrete network of finite populations by a Markov chain. In order to determine finite-size effects—deviations from the mean-field limit due to the finite size of the populations in the network—we analyze the fluctuations of this Markov chain and set up an approximating system of diffusion processes. We show that a well-posed stochastic neural field equation with a noise term accounting for finite-size effects on traveling wave solutions is obtained as the strong continuum limit.

## Introduction

The analysis of networks of neurons of growing size quickly becomes involved from a computational as well as from an analytic perspective when one tracks the spiking activity of every neuron in the network. It can therefore be useful to zoom out from the microscopic perspective and identify a population activity as an average over a certain group of neurons. In the heuristic derivation of such population models it is usually assumed that each of the populations in the network is infinite, such that, in the spirit of the law of large numbers, the description of the activity in each population reduces to a description of the mean. By considering a spatially extended network and letting the density of populations go to infinity, neural field equations are obtained as the continuum limit of these models. Here we consider the voltage-based neural field equation, which is a nonlocal evolution equation of the form

1 $$ \frac{\partial}{\partial t} u(x,t) = -u(x,t) + w\ast F\bigl(u(\cdot, t) \bigr) (x),\quad x\in \mathbb {R}, t\geq0, $$

where $u(x,t)$ describes the average membrane potential in the population at *x* at time *t*, $w:\mathbb {R}\rightarrow[0,\infty)$ is a kernel describing the strengths of the synaptic connections between the populations, and the gain function $F:\mathbb {R}\rightarrow[0, 1]$ relates the potential to the activity in the population.

Neural field equations were first introduced by Amari [1] and Wilson and Cowan [2, 3] and have since been used extensively to study the spatio-temporal dynamics of the activity in coupled populations of neurons. While they are of a relatively simple form, they exhibit a variety of interesting spatio-temporal patterns. For an overview see for example [4–8]. In this article we will concentrate on traveling wave solutions, modeling the propagation of activity, which were proven to exist in [9].

The communication of neurons is subject to noise. It is therefore crucial to study stochastic versions of (1). While several sources of noise have been identified on the single neuron level, it is not clear how noise translates to the level of populations. Since neural field equations are derived as mean-field limits, the usual effects of noise should have averaged out on this level. However, the actual finite size of the populations leads to deviations from the mean-field behavior, suggesting finite-size effects as an intrinsic source of noise.

The (heuristic) derivation of neural field equations involves two approximation steps. First, the local dynamics in each population is reduced to a description of the mean activity. Second, the discrete network is approximated by a continuum. In this article we make these two approximation steps explicit. In order to describe deviations from the mean-field behavior for finite population sizes, we set up a Markov chain to describe the evolution of the activity in the finite network, extending a model by Bressloff and Newby [10]. The transition rates are chosen in such a way that we obtain the voltage-based neural network equation in the infinite-population limit. We analyze the fluctuations of the Markov chain in order to determine a stochastic correction term describing finite-size effects. In the case of fluctuations around traveling wave solutions, we set up an approximating system of diffusion processes and prove that a well-posed stochastic neural field equation is obtained in the continuum limit.

In order to derive corrections to the neural field equation accounting for finite-size effects, in [11], Bressloff (following Buice and Cowan [12]) sets up a continuous-time Markov chain describing the evolution of the activity in a finite network of populations of finite size *N*. The rates are chosen such that in the limit as $N \rightarrow\infty$ one obtains the usual activity-based network equation. He then carries out a van Kampen system size expansion of the associated master equation in the small parameter $1/N$ to derive deterministic corrections of the neural field equation in the form of coupled differential equations for the moments. To first order, the finite-size effects can be characterized as Gaussian fluctuations around the mean-field limit.

The model is considered from a mathematically rigorous perspective by Riedler and Buckwar in [13]. They make use of limit theorems for Hilbert-space valued piecewise deterministic Markov processes recently obtained in [14] as an extension of Kurtz’s convergence theorems for jump Markov processes to the infinite-dimensional setting. They derive a law of large numbers and a central limit theorem for the Markov chain, realizing the double limit (number of neurons per population to infinity and continuum limit) at the same time. They formally set up a stochastic neural field equation, but the question of well-posedness is left open.

In [10], Bressloff and Newby extend the original approach of [11] by including synaptic dynamics and consider a Markov chain modeling the activity coupled to a piecewise deterministic process describing the synaptic current (see also Sect. 6.4 in [8] for a summary). In two different regimes, the model covers the case of Gaussian-like fluctuations around the mean-field limit as derived in [11], as well as a situation in which the activity has Poisson statistics as considered in [15].

Here we consider the question how finite-size effects can be included in the voltage-based neural field equation. We take up the approach of describing the dynamics of the activity in a finite-size network by a continuous-time Markov chain and motivate a choice of jump rates that will lead to the voltage-based network equation in the infinite-population limit. We derive a law of large numbers and a central limit theorem for the Markov chain. Instead of realizing the double limit as in [13], we split up the limiting procedure, which in particular allows us to insert further approximation steps. We follow the original approach by Kurtz to determine the limit of the fluctuations of the Markov chain. By linearizing the noise term around the traveling wave solution, we obtain an approximating system of diffusion processes. After introducing correlations between populations lying close together (cf. Sect. 5.1) we obtain a well-posed $L^{2}(\mathbb {R})$-valued stochastic evolution equation, with a noise term approximating finite-size effects on traveling waves, which we prove to be the strong continuum limit of the associated network. Further results concerning (stochastic) stability of travelling waves and a corresponding multiscale analysis for the stochastic neural field equations derived in this paper are contained in the references [16, 17], see also the PhD-thesis [18].

The article is structured as follows. We recall how population models can be derived heuristically in Sect. 2 and summarize the work on the description of finite-size effects that can be found in the literature so far. In Sect. 3 we introduce our Markov chain model for determining finite-size effects in the voltage-based neural field equation and prove a law of large numbers and a central limit theorem for our choice of jump rates. We use it to set up a diffusion approximation with a noise term accounting for finite-size effects on traveling wave solutions in Sect. 4. Finally, in Sect. 5, we prove that a well-posed stochastic neural field equation is obtained in the continuum limit.

### Assumptions on the Parameters

As usual, we take the gain function $F:\mathbb {R}\rightarrow[0,1]$ to be a sigmoid function, for example $F(x) = \frac{1}{1+ e^{-\gamma(x-\kappa)}}$ for some $\gamma> 0$, $0<\kappa<1$. In particular we assume that

(i) $F \geq0$, $\lim_{x \downarrow-\infty}F(x)=0$, $\lim_{x\uparrow \infty}F(x)=1$,
(ii) $F(x)-x$ has exactly three zeros $0 < a_{1} < a < a_{2}<1$,
(iii) $F \in\mathcal{C}^{3}$ and $F'$, $F''$ and $F'''$ are bounded,
(iv) $F' > 0$, $F'(a_{1}) < 1$, $F'(a_{2}) < 1$, $F'(a)>1$.

Our assumptions on the synaptic kernel *w* are the following:

(i) $w \in\mathcal{C}^{1}$,
(ii) $w(x,y)=w(|x-y|)\geq0$ is nonnegative and homogeneous,
(iii) $\int_{-\infty}^{\infty} w(x) \,dx = 1$, $w_{x} \in L^{1}$.

Assumption (iv) on *F* implies that $a_{1}$ and $a_{2}$ are stable fixed points of (1), while *a* is an unstable fixed point. It has been shown in [9] that under these assumptions there exists a unique monotone traveling wave solution to (1) connecting the stable fixed points (and, in [19], that traveling wave solutions are necessarily monotone). That is, there exist a unique wave profile $\hat{u}:\mathbb {R}\rightarrow [0,1]$ and a unique wave speed $c \in \mathbb {R}$ such that $u^{\mathrm{TW}}(t,x) = u^{\mathrm{TW}} _{t}(x) := \hat{u}(x-ct)$ is a solution to (1), i.e.

$$\begin{aligned} -c\partial_{x} u^{\mathrm{TW}}_{t} (x) &= \partial_{t} u^{\mathrm{TW}}_{t}(x) = -u^{\mathrm{TW}}_{t}(x) + \int _{-\infty}^{\infty} w(x-y) F\bigl(u^{\mathrm{TW}}_{t}(y) \bigr) \,dy \\ & = -u^{\mathrm{TW}}_{t}(x) + w \ast F\bigl(u^{\mathrm{TW}}_{t} \bigr) (x), \end{aligned}$$

and

$$\lim_{x \rightarrow-\infty} \hat{u}(x) = a_{1},\qquad \lim _{x\rightarrow \infty} \hat{u}(x) = a_{2}. $$

As also pointed out in [9], we can without loss of generality assume that $c\geq0$. Note that $\hat{u}_{x} \in L^{2}(\mathbb {R})$ since in the case $c>0$

$$\begin{aligned} \int\hat{u}_{x}^{2}(x) \,dx & = \int\hat{u}_{x}(x) \frac{1}{c} \bigl( \hat {u}(x) - w \ast F(\hat{u}) (x) \bigr) \,dx \\ & \leq\frac{1}{c} \biggl( \|\hat{u}\|_{\infty} + \|F \|_{\infty} \int w(x) \,dx \biggr) \int\hat{u}_{x}(x) \,dx = \frac{1}{c}( a_{2}+1) (a_{2}-a_{1}), \end{aligned}$$

and in the case $c=0$

$$\int\hat{u}_{x}^{2}(x) \,dx = \int\hat{u}_{x}(x) w_{x} \ast F(\hat{u}) (x) \,dx \leq\|w_{x}\|_{1} (a_{2}-a_{1}). $$

## Finite-Size Effects in Population Models

### Population Models

In population models, or firing rate models, instead of tracking the spiking activity of every neuron in the network, neurons are grouped together and the activity is identified as a population average. We start by giving a heuristic derivation of population models, distinguishing as usual between an activity-based and a voltage-based regime.

We consider a population of *N* neurons. We say that a neuron is ‘active’ if it is in the process of firing an action potential such that its membrane potential is larger than some threshold value *κ*. If Δ is the width of an action potential, then a neuron is active at time *t* if it fired a spike in the time interval $(t-\Delta,t]$. We define the population activity at a given time *t* as the proportion of active neurons,

2 $$ a^{N}(t) = \frac{\mbox{\# neurons that are active at time }t}{N} \in\biggl\{ 0, \frac{1}{N}, \frac{2}{N}, \ldots, 1\biggr\} . $$

We assume that all neurons in the population are identical and receive the same input. If the neurons fire independently from each other, then, for a constant input current *I*,

$$ a^{N}(t) \xrightarrow{N\rightarrow\infty} F(I), $$

where $F(I)$ is the probability that a neuron receiving constant stimulation *I* is active. In the infinite-population limit, the population activity is thus related to the input current via the function *F*, called the *gain function*. Sometimes one also defines *F* as a function of the potential *u*, assuming that the potential is proportional to the current as in Ohm’s law. *F* is typically a nonlinear function. It is usually modeled as a sigmoid, for example

$$F(x) = \frac{1}{1+e^{-\gamma(x-\kappa)}} $$

for some $\gamma>0$ and some threshold $\kappa>0$, imitating the threshold-like nature of spiking activity.

Sometimes a *firing rate* is considered instead of a probability. We define the population firing rate $\lambda^{N}$ as

$$\lambda^{\delta,N}(t) = \frac{\mbox{\# spikes in the time interval }(t-\delta,t]}{\delta N}. $$

If $\delta=\Delta$, then $\Delta\lambda^{\delta,N}(t)=a^{N}(t)$. At constant potential *u*, $\lim_{\delta\rightarrow0} \lim_{N\rightarrow\infty} \lambda^{\delta ,N}(t) = \lambda(u)$, where $\lambda(u)$ is the single neuron firing rate. Note that $\lambda\leq\frac{1}{\Delta}$. The firing rate is related to the probability $F(u)$ via

$$F(u) \approx\lambda(u) \Delta. $$

If the stimulus varies in time, then the activity may track this stimulus with some delay such that

$$a(t+\tau_{a}) = F\bigl(u(t)\bigr) $$

for some time constant $\tau_{a}$. Taylor expansion of the left-hand side gives an approximate description of the (infinite-population) activity in terms of the differential equation

3 $$ \tau_{a} \dot{a}(t) = -a(t)+F\bigl(u(t)\bigr), $$

to which we refer as the *rate equation*.

We now consider a network of *P* populations, each consisting of *N* neurons. We assume that each presynaptic spike in population *j* at time *s* causes a postsynaptic potential

$$h(t-s)= \frac{1}{N} w_{ij} \frac{1}{\tau_{m}} e^{-\frac{1}{\tau_{m}}(t-s)} $$

in population *i* at time *t*. Here the $(w_{ij})$ are weights characterizing the strength of the synaptic connections between populations *i* and *j*, and $\tau_{m}$ is the membrane time constant, describing how fast the membrane potential relaxes back to its resting value.

Under the assumption that all inputs add up linearly, the potential in population *i* at time *t* is given as

$$ u_{i}^{N}(t) = \sum_{j=1}^{P} w_{ij} \int_{-\infty}^{t} \frac{1}{\tau_{m}} e^{-\frac{1}{\tau_{m}}(t-s)} a_{j}^{N}(s) \,ds , $$

or, in differential form,

4 $$ \tau_{m}\dot{u}_{i}^{N}(t) = - u_{i}^{N} (t) + \sum_{j=1}^{P} w_{ij} a_{j}^{N}(t) . $$

In the infinite-population limit we therefore obtain the coupled system

5 $$\begin{aligned} \begin{aligned} \tau_{m}\dot{u}_{i} (t) & = - u_{i} (t) + \sum_{j=1}^{P} w_{ij} a_{j} (t), \\ \tau_{a}\dot{a}_{i} (t) & = - a_{i} (t) + F \bigl(u_{i}(t)\bigr) ,\quad i = 1, \ldots P . \end{aligned} \end{aligned}$$

The behavior of (5) depends on the two time constants, $\tau_{m}$ and $\tau_{a}$. Two different regimes can be identified in which the model can be reduced to just one of the two variables, *u* or *a*.

#### Case 1: $\tau_{m} \gg\tau_{a} \rightarrow0$

Setting $\tau_{a} = 0$ yields $a_{i} (t) = F(u_{i} (t))$ and thus (5) reduces to

6 $$ \tau_{m} \dot{u}_{i}(t) = - u_{i} (t) + \sum_{j=1}^{P} w_{ij} F \bigl(u_{j}(t)\bigr) , $$

which we will call the *voltage-based neural network equation*.

#### Case 2: $\tau_{a} \gg\tau_{m} \rightarrow0$

Setting $\tau_{m} = 0$ yields $u_{i} (t) = \sum_{j=1}^{P} w_{ij} a_{j} (t)$ and thus (5) reduces in this case to

7 $$ \tau_{a} \dot{a}_{i}(t) = - a_{i} (t) + F \Biggl( \sum_{j=1}^{P} w_{ij} a_{j} (t) \Biggr) , $$

which we will call the *activity-based neural network equation*.

Throughout this paper we will be interested in the former regime of the voltage-based neural network equation, thereby assuming $\tau_{a}$ small. Our aim is to derive stochastic differential equations in a first step for the infinite-population limit $N\to\infty $ and subsequently for the continuum limit $P\to\infty$, describing the asymptotic fluctuations.

### Finite-Size Effects

The rate equation discussed in the previous subsection depends crucially on the way, how activity is measured. Starting from the definition (2) for given $\delta> 0$, the value will depend on the relation between the value of *δ* and the width Δ of an action potential. In [10], a model for the evolution of the activity in a network of finite populations is set up, based on the definition

$$a^{\delta, N}_{j}(t) = \frac{\mbox{\# spikes in }(t-\delta, t]\mbox{ in population }j}{\delta N } $$

of the activity in population *j*. Here, *δ* is a time window of variable size. If *δ* is chosen as the width of an action potential Δ, one obtains the original notion of the activity, $\Delta a^{\Delta, N} = a^{N}$. Note that the number of spikes in the time interval $(t-\delta,t]$ is limited by $n_{\mathrm{max}}:= N \vee [\frac{\delta N}{\Delta} ] $.

The dynamics of $a^{\delta, N}$ is modeled as a Markov chain with state space $\{ 0, \frac{1}{\delta N}, \ldots , \frac{n_{\mathrm{max}}}{\delta N} \}^{P}$ and jump rates

8 $$\begin{aligned} \begin{aligned} q_{a}^{N} \biggl(x, x+ \frac{1}{\delta N} e_{i}\biggr) & = \frac{1}{\tau_{a}} \delta N \lambda\bigl(u^{N}_{i}(t)\bigr) \quad \mbox{if }x(i) < \frac{n_{\mathrm{max}}}{\delta N}, \\ q_{a}^{N}\biggl(x, x- \frac{1}{\delta N} e_{i} \biggr) & = \frac{1}{\tau_{a}} \delta N x (i) . \end{aligned} \end{aligned}$$

Here $e_{i}$ denotes the *i*th unit vector, $\lambda(u)$ is the firing rate at potential *u*, related to the probability $F(u)$ via $\Delta \lambda(u) = F(u)$, and $u^{N}$ evolves according to (4)

$$\dot{u}_{i}^{N}(t) = \frac{1}{\tau_{m}} \Biggl( -u_{i}^{N} (t) + \sum_{j=1}^{P} w_{ij} a_{j}^{N}(t) \Biggr) . $$

The idea for these transition rates is that the activation rate should be proportional to $\lambda(u)$, while the inactivation rate should be proportional to the activity itself.

The following two regimes are then considered in [10].

#### Case 1: $\delta=1$, $\tau_{a} \gg\tau_{m} \rightarrow0$

In the first regime, the size of the time window *δ* is fixed, say $\delta=1$. If $\tau_{a} \gg\tau_{m} \rightarrow0$, then as in Sect. 2.1, $u^{N}_{i}(t) = \sum_{j=1}^{P} w_{ij} a_{j}^{\delta, N}(t)$. The description of the Markov chain can thus be closed in the variables $a^{\delta, N}_{i}$, leading to the model already considered in [11]. In the limit $N\rightarrow \infty$ one obtains the activity-based network equation

9 $$ \tau_{a} \dot{a}_{i}^{N}(t) = -a_{i}^{N}(t) + \lambda \Biggl( \sum _{j=1}^{P} w_{ij} a^{N}_{j}(t) \Biggr). $$

By formally approximating to order $\frac{1}{N}$ in the associated master equation, they derive a stochastic correction to (9), leading to the diffusion approximation

$$\begin{aligned} da^{\delta, N}_{i}(t) \approx&\frac{1}{\tau_{a}} \Biggl( - a^{\delta, N}_{i}(t) + \lambda \Biggl( w_{ij} \sum _{j=1}^{P} a^{\delta, N}_{j}(t) \Biggr) \Biggr) \,dt \\ &{} + \frac{1}{\sqrt{\tau_{a} N}} \Biggl( a^{\delta, N}_{i}(t) + \lambda \Biggl( \sum_{j=1}^{P} w_{ij} a^{\delta, N}_{j}(t) \Biggr) \Biggr)^{\frac{1}{2}} \,dB_{j}(t) \end{aligned}$$

for independent Brownian motions $B_{j}$.

In [13], Riedler and Buckwar rigorously derive a law of large numbers and a central limit theorem for the sequence of Markov chains as *N* tends to infinity. Note that the nature of the jump rates is such that the process has to be ‘forced’ to stay in its natural domain $[0,\frac{n_{\mathrm{max}}}{N}]$ by setting the jump rate to 0 at the boundary. As they point out, this discontinuous behavior is difficult to deal with mathematically. They therefore have to slightly modify the model and allow the activity to be larger than $\frac{n_{\mathrm{max}}}{N}$. They embed the Markov chain into $L^{2}(D)$ for a bounded domain $D\subset \mathbb {R}^{d}$ and derive the LLN in $L^{2}(D)$ and the CLT in the Sobolev space $H^{-\alpha}(D)$ for some $\alpha>d$.

#### Case 2: $\delta=\frac{1}{N}$, $\tau_{m} \gg\tau_{a}$

In the second regime, the size of the time window *δ* goes to 0 as *N* goes to infinity such that $\delta N=1$. In this case,

$$a^{\delta, N}_{i}(t) = \frac{\mbox{\# spikes in }(t-\delta,t]\mbox{ in pop. } i}{\delta N} \approx \frac{\lambda(u_{i}^{N}(t)) \delta N}{\delta N}=\lambda\bigl(u_{i}^{N}(t)\bigr). $$

The corresponding Markov chain has state space $\{ 0, 1, 2, \ldots, n_{\mathrm{max}}\}^{P}$. Since $\tau_{a} \ll\tau_{m}$, changes in $u_{i} (t)$ are slow in comparison to changes of the activity, and for fixed voltage *u*, the Markov chain in fact has a stationary distribution that is approximately Poisson with rate $\lambda(u)$ (see e.g. [10]). This corresponds to the regime considered in [15].

Note that we cannot expect to have a deterministic limit for the activity as $N\to\infty$ in this case, so we do not have a deterministic limit for the voltage. We will therefore consider a third regime with $\delta> 0$ fixed in this paper and study its asymptotic behavior in the large population limit $N\to\infty$.

#### Case 3: $\delta=\Delta$, $\tau_{m} \gg\tau_{a} \rightarrow 0$

We can expect in this case to have a law of large numbers, i.e. a deterministic limit for the activity. Since $a_{i} (t) \approx F(u_{i} (t))$ we also obtain a law of large numbers for the voltage. In order to derive an appropriate diffusion approximation, we will have to reconsider the transition rates of the Markov chain (8).

Going back to our original definition of the activity let us fix the time window *δ* to be the length of an action potential Δ. We assume that the potential evolves slowly, $\tau_{m} \gg0$. Speeding up time, we define

$$\tilde{u}_{i}^{N}(t) = u_{i}^{N}(t \tau_{m}). $$

Then

$$\begin{aligned} \tilde{u}_{i}^{N}(t) & = \sum_{j=1}^{P} w_{ij} \int_{-\infty}^{t\tau_{m}} \frac{1}{\tau_{m}} e^{-\frac{1}{\tau_{m}}(t\tau_{m} -s)} a_{j}^{N}(s) \,ds \\ & = \sum_{j=1}^{P} w_{ij} \int_{-\infty}^{t} e^{-(t-s)} a_{j}^{N}(s \tau_{m}) \,ds. \end{aligned}$$

For some large *n*,

$$ \tilde{u}_{i}^{N}(t) \approx\sum _{j=1}^{P} w_{ij} \sum _{k=-\infty}^{[tn]-1} e^{-(t-\frac {k}{n})} \int_{\frac{k}{n}}^{\frac{k+1}{n}} a_{j}^{N}(s \tau_{m}) \,ds. $$

The potentials $\tilde{u}_{i}^{N}$ therefore only depend on the time-averaged activities given for ${\frac{k}{n}\leq t < \frac {k+1}{n}}$ as

$$\tilde{a}_{i}^{N}(t) = n \int_{\frac{k}{n}}^{\frac{k+1}{n}} a_{i}^{N}(s \tau_{m}) \,ds. $$

We have

$$ \tilde{u}_{i}^{N}(t) \approx\sum _{j=1}^{P} w_{ij} \sum _{k=-\infty}^{[tn]-1}\frac{1}{n} e^{-(t-\frac{k}{n})} \tilde{a}_{j}^{N}\biggl(\frac{k}{n}\biggr) \approx\sum _{j=1}^{P} w_{ij} \int_{-\infty}^{t} e^{-(t-s)} \tilde{a}_{j}^{N}(s) \,ds. $$

Since the relaxation rate $\tau_{a}$ approaches zero, the time-averaged activity $\tilde{a}_{i}^{N}(t)$ approximates $F(\tilde{u}^{N}_{i} (t))$ and thus

10 $$ \tilde{a}^{N}_{i} (t) \approx F\bigl( \tilde{u}^{N}_{i} (t)\bigr) \approx F \Biggl( \sum _{j=1}^{P} w_{ij} \int_{-\infty}^{t} e^{-(t-s)} \tilde{a}_{j}^{N}(s) \,ds \Biggr) , $$

with equality in the limit $N\to\infty$. Differentiating (10) w.r.t. *t* therefore yields

11 $$\begin{aligned} \frac{d}{dt} \tilde{a}_{i}(t) & = \frac{d}{dt} F\bigl( \tilde{u}_{i}(t)\bigr) \\ & = F'\bigl(\tilde{u}_{i}(t)\bigr) \Biggl(- \tilde{u}_{i}(t) + \sum_{j=1}^{P} w_{ij} F\bigl(\tilde{u}_{j}(t)\bigr) \Biggr) \\ & = F'\bigl(F^{-1}\bigl(\tilde{a}_{i}(t) \bigr)\bigr) \Biggl( - F^{-1}\bigl(\tilde{a}_{i}(t)\bigr) + \sum_{j=1}^{P} w_{ij} \tilde{a}_{j}(t) \Biggr). \end{aligned}$$

If $N <\infty$, then the finite size of the populations causes deviations from (11). In order to determine these finite-size effects, in the next section we will set up a Markov chain $X^{P,N}$ to describe the evolution of the time-averaged activity $\tilde{a}^{N}$.

## A Markov Chain Model for the Activity

We will model the time-averaged activity by a Markov chain $X^{P,N}$ on the state space $E^{P,N} = \{0, \frac{1}{N}, \frac{2}{N}, \ldots, 1\} ^{P}$. To this end consider the following jump rates:

12 $$\begin{aligned} \begin{aligned} q^{P,N}\biggl(x, x+ \frac{1}{N}e_{i}\biggr) & = N F' \bigl(F^{-1}(x_{i})\bigr) \Biggl(-F^{-1}(x_{i})+ \sum_{j=1}^{P} w_{ij} x_{j} \Biggr)_{+}, \\ q^{P,N}\biggl(x, x-\frac{1}{N}e_{i}\biggr) & = N F'\bigl(F^{-1}(x_{i})\bigr) \Biggl(- F^{-1}(x_{i})+\sum_{j=1}^{P} w_{ij} x_{j} \Biggr)_{-}, \end{aligned} \end{aligned}$$

for $x\in E^{P,N}$ with $x_{i}\in\{\frac{1}{N}, \ldots, 1-\frac{1}{N}\}$. Here, we used the notation $x_{+} := x\vee0$, $x_{-} := (-x) \vee0$, and $e_{i}$ denotes the *i*th unit vector.

Note that, for $N\in\mathbb{N}$ large, the interior $(E^{P,N})^{\circ}:= \{\frac{1}{N}, \ldots, 1-\frac{1}{N}\}^{P}$ will be invariant, hence the Markov chain, when started in $(E^{P,N})^{\circ}$ stays away from the boundary. Indeed, for large $N\in\mathbb{N}$ it follows that

$$q^{P,N} \biggl(x, x+\frac{1}{N} e_{i}\biggr) = 0 \qquad \biggl(\mbox{resp. } q^{P,N} \biggl(x, x-\frac{1}{N} e_{i}\biggr) = 0 \biggr) $$

for $x\in(E^{P,N})^{\circ}$ with $x_{i} = 1-\frac{1}{N}$ (resp. $x_{i} = \frac{1}{N}$), because $F^{-1} (1-\frac{1}{N}) > 1-\frac{1}{N} \ge\sum_{j=1}^{P} w_{ij} x_{j}$, hence $q(x, x+\frac{1}{N} e_{i}) = 0$, (resp. $F^{-1} (\frac{1}{N}) < \frac{1}{N} \le\sum_{j=1}^{P} w_{ij} x_{j}$, hence $q(x, x-\frac{1}{N} e_{i}) = 0$) for large *N*, using the limiting behavior $\lim_{x \uparrow1} F^{-1}(x) = \infty$ (resp. $\lim_{x \downarrow0} F^{-1}(x) = - \infty$).

The idea behind the choice of our rates is the following: the time-averaged activity tends to jump up (down) if the potential in the population, which is approximately given by $F^{-1}(x_{i})$, is lower (higher) than the input from the other populations, which is given by $\sum_{j=1}^{P} w_{ij} x_{j}$. The probability that the activity jumps down (up) when the potential is lower (higher) than the input is assumed to be negligible. The jump rates are proportional to the difference between the two quantities, scaled by the factor $F'(F^{-1}(x_{i}))$. They are therefore higher in the sensitive regime where $F'\gg1$, that is, where small changes in the potential have large effects on the activity. If $a^{N}_{i} = F(u^{N}_{i})$ in all populations *i*, then the system is in balance.

We will see in Proposition 1 below that the Markov chain converges to the solution of (11) for increasing population size $N\uparrow\infty$.

In [11] a different choice of jump rates was suggested in analogy to (8):

$$\begin{aligned} \tilde{q}\biggl(x, x+\frac{1}{N}e_{i}\biggr) & = N F'\bigl(F^{-1}(x_{i})\bigr) \sum _{j=1}^{P} w_{ij} x_{j}, \\ \tilde{q}\biggl(x, x-\frac{1}{N}e_{i}\biggr) & = N F'\bigl(F^{-1}(x_{i})\bigr) F^{-1}(x_{i}). \end{aligned}$$

Also this choice leads to (11) in the limit. With these rates, however, the jump rates are high in regions where the activity is high. Since, as explained above, one should think of the Markov chain as governing a slowly varying time-averaged activity, (12) seems like a more natural choice.

The generator of $Q^{P,N}$ of $X^{P,N}$ is given for bounded measurable $f:E^{P,N} \rightarrow \mathbb {R}$ by

$$\begin{aligned} Q^{P,N}f (x) =& N \sum_{k=1}^{P} F'\bigl(F^{-1}(x_{k})\bigr) \Biggl( \Biggl(-F^{-1}(x_{k}) + \sum_{j=1}^{P} w_{kj} x_{j} \Biggr)_{+} \\ &{}\times\biggl(f\biggl(x+ \frac {1}{N}e_{k}\biggr)-f(x)\biggr) \\ &{} {} + \Biggl(-F^{-1}(x_{k}) + \sum _{j=1}^{P} w_{kj} x_{j} \Biggr)_{-} \biggl(f\biggl(x-\frac{1}{N}e_{k}\biggr) - f(x)\biggr) \Biggr). \end{aligned}$$

Let $N_{0}$ be such that for $N\ge N_{0}$ the interior $(E^{P,N})^{\circ}$ is an invariant set for $X^{P,N}$.

The jump rates out of the interval $[\frac{1}{N_{0}}, 1-\frac {1}{N_{0}} ]$ are 0.

### Proposition 1

*Let* $X^{P}$ *be the* (*deterministic*) *Feller process on* $[1/N_{0}, 1-1/N_{0}]^{P}$ *with generator*

$$L^{P}f(x) = \sum_{k=1}^{P} F'\bigl(F^{-1}(x_{k})\bigr) \Biggl( - F^{-1}(x_{k}) + \sum_{j=1}^{P} w_{kj} x_{j} \Biggr) \partial_{k}f(x). $$

*If* $X^{P,N}(0) \xrightarrow[N\rightarrow\infty]{d} X^{P}(0) $, *then* $X^{P,N} \xrightarrow[N \rightarrow\infty]{d} X^{P}$ *on the space of càdlàg functions* $D([0, \infty),[1/N_{0},1-1/N_{0}]^{P})$ *with the Skorohod topology* (*where* $\xrightarrow{d}$ *denotes convergence in distribution*).

### Proof

By a standard theorem on the convergence of Feller processes (cf. [20], Thm. 19.25) it is enough to prove that for ${f \in \mathcal{C}^{\infty}([1/N_{0},1-1/N_{0}]^{P})}$ there exist bounded measurable $f_{N}$ such that ${\|f_{N} - f\|_{\infty} \xrightarrow {N\rightarrow\infty} 0}$ and $\|Q^{P,N}f_{N} - L^{P}f\|_{\infty} \xrightarrow{N \rightarrow\infty} 0$.

Let thus ${f \in\mathcal{C}^{\infty}([1/N_{0},1-1/N_{0}]^{P})}$ and set ${f_{N}(x) = f (\frac{[x_{1}N]}{N}, \ldots,\frac{[x_{P}N]}{N} )}$. Then it is easy to see that

$$Q^{P,N}f_{N}(x) \xrightarrow{N\rightarrow\infty} L^{P}f(x) $$

uniformly in *x*. □

## Diffusion Approximation

We are now going to approximate $X^{P,N}$ by a diffusion process. To this end, we follow the standard approach due to Kurtz and derive a central limit theorem for the fluctuations of $X^{P,N}$. This will give us a candidate for a stochastic correction term to (11).

### A Central Limit Theorem

The general theory of continuous-time Markov chains implies the following semi-martingale decomposition:

$$X_{k}^{P,N}(t) = X_{k}^{P,N}(0) + \int_{0}^{t} Q^{P,N} \pi_{k} \bigl(X^{P,N}(s)\bigr) \,ds + M_{k}^{P,N}(t), $$

where $\pi_{k}: (0,1)^{P} \rightarrow(0,1)$, $x \mapsto x_{k}$, is the projection onto the *k*th coordinate, $Q^{P,N} \pi_{k} (X^{P,N} (s)) = \sum_{y\in E^{P,N}} q^{P,N} ( X^{P,N} (s), y ) y_{k}$ is the (signed) kernel $Q^{P,N}$ applied to the function $\pi_{k}$ evaluated at the state $X^{P,N} (s)$, and

$$M_{k}^{P,N}(t) := X_{k}^{P,N}(t) - X_{k}^{P,N}(0) - \int_{0}^{t} Q^{P,N} \pi_{k} \bigl(X^{P,N}(s)\bigr) \,ds $$

is a zero mean square-integrable martingale (with right-continuous sample paths having left limits in $t> 0$). Moreover,

$$\bigl( M_{k}^{P,N}(t) \bigr)^{2} - \int_{0}^{t} \sum_{y\in E^{P,N}} q^{P,N} \bigl( X_{k}^{P,N}(s),y \bigr) \bigl(X^{P,N}_{k} (s) - y_{k} \bigr)^{2} \,ds $$

is a martingale too (see [21]). The general theory of stochastic processes states that for any square-integrable right-continuous martingale $M_{t}$, $t\ge0$, having left limits in $t > 0$, there exists a unique increasing previsible process $\langle M\rangle_{t}$ starting at 0, such that $M_{t}^{2} - \langle M\rangle_{t}$, $t\ge0$, is a martingale too. We can therefore conclude that

$$\bigl\langle M^{P,N}\bigr\rangle _{t} = \int_{0}^{t} \sum_{y\in E^{P,N}} q^{P,N} \bigl( X_{k}^{P,N}(s),y \bigr) \bigl(X^{P,N}_{k} (s) - y_{k} \bigr)^{2} \,ds . $$

The bracket process will be crucial to determine the limit of the fluctuations as seen in the next proposition.

#### Proposition 2

$$\begin{aligned} &\bigl(\sqrt{N} M^{P,N}_{k} \bigr) \\ &\quad \xrightarrow[N\rightarrow \infty]{d} \Biggl( \int_{0}^{t} \Biggl(F' \bigl(F^{-1}\bigl(X_{k}^{P}(s)\bigr)\bigr) \Biggl\vert -F^{-1}\bigl(X_{k}^{P}(s)\bigr) + \sum _{j=1}^{P} w_{kj}X^{P}_{j}(s) \Biggr\vert \Biggr)^{\frac{1}{2}} \,dB_{k}(s) \Biggr) \end{aligned}$$

*on* $\mathcal{D}([0,\infty), \mathbb {R}^{P})$, *where* *B* *is a* *P*-*dimensional standard Brownian motion*, *and* $X^{P}$ *is the Feller process from Proposition* 1.

#### Proof

Using

$$\begin{aligned} \bigl\langle M^{P,N}_{k} \bigr\rangle _{t} =& \int_{0}^{t} \sum_{y\in E^{P,N}} q^{P,N}\bigl(X^{P,N}(s), y\bigr) \bigl(X_{k}^{P,N}(s) - y_{k}\bigr)^{2} \,ds \\ = &\frac{1}{N} \int_{0}^{t} F'\bigl(F^{-1} \bigl(X^{P,N}_{k}(s)\bigr)\bigr) \Biggl( \Biggl(-F^{-1} \bigl(X^{P,N}_{k}(s)\bigr) + \sum _{j=1}^{P} w_{kj} X^{P,N}_{j}(s) \Biggr)_{+} \\ & {} + \Biggl(-F^{-1}\bigl(X^{P,N}_{k}(s)\bigr) + \sum_{j=1}^{P} w_{kj} X^{P,N}_{j}(s) \Biggr)_{-} \Biggr) \,ds \\ =& \frac{1}{N} \int_{0}^{t} F'\bigl(F^{-1} \bigl(X^{P,N}_{k}(s)\bigr)\bigr) \Biggl\vert -F^{-1}\bigl(X^{P,N}_{k}(s)\bigr) + \sum _{j=1}^{P} w_{kj} X^{P,N}_{j}(s) \Biggr\vert \,ds. \end{aligned}$$

Thus,

$$\bigl\langle \sqrt{N}M_{k}^{P,N} \bigr\rangle _{t} \xrightarrow{N\rightarrow \infty} \int_{0}^{t} F'\bigl(F^{-1} \bigl(X^{P}_{k}(s)\bigr)\bigr) \Biggl\vert -F^{-1}\bigl(X^{P}_{k}(s)\bigr) + \sum _{j=1}^{P} w_{kj} X^{P}_{j}(s) \Biggr\vert \,ds $$

in probability. For $k \neq l$,

$$ \bigl\langle M^{P,N}_{k}, M^{P,N}_{l} \bigr\rangle _{t} = \int_{0}^{t} \sum_{y} q^{P,N}\bigl(X^{P,N}(s), y\bigr) \bigl(X_{k}^{P,N}(s) - y_{k} \bigr) \bigl(X_{l}^{P,N}(s) - y_{l}\bigr) \,ds = 0, $$

since for *y* with $q^{P,N}(X^{P,N}(s),y) > 0$ at least one of $X_{k}^{P,N}(s) - y_{k}$ and $X_{l}^{P,N}(s) - y_{l}$ is always 0. Now

$$E \Bigl(\sup_{t} \sqrt{N} \bigl\Vert M^{P,N}(t)-M^{P,N}(t-) \bigr\Vert _{2} \Bigr) \leq\frac {1}{\sqrt{N}} \xrightarrow{N \rightarrow\infty}0, $$

and the statement follows by the martingale central limit theorem; see for example Theorem 1.4, Chap. 7 in [22]. □

This suggests to approximate $X^{P,N}$ by the system of coupled diffusion processes

$$\begin{aligned} &da_{k}^{P,N}(t) \\ &\quad = F'\bigl(F^{-1} \bigl(a^{P,N}_{k}(t)\bigr)\bigr) \Biggl( - F^{-1} \bigl(a^{P,N}_{k}(t)\bigr) + \sum _{j=1}^{P} w_{kj} a^{P,N}_{j}(t) \Biggr) \,dt \\ &\qquad {} + \frac{1}{\sqrt{N}} \Biggl(F'\bigl(F^{-1} \bigl(a^{P,N}_{k}(t)\bigr)\bigr) \Biggl\vert -F^{-1}\bigl(a^{P,N}_{k}(t)\bigr) + \sum _{j=1}^{P} w_{kj} a^{P,N}_{j}(t) \Biggr\vert \Biggr)^{\frac{1}{2}} \,dB_{k}(t), \end{aligned}$$

$1 \leq k \leq P$.

Using Itô’s formula, we formally obtain an approximation for $u^{P,N}_{k} := F^{-1}(a_{k}^{P,N})$,

13 $$\begin{aligned} \begin{aligned}[b] du^{P,N}_{k}(t) ={}& \Biggl( - u^{P,N}_{k}(t) + \sum_{j=1}^{P} w_{kj} F \bigl(u^{P,N}_{j}(t)\bigr) \\ & {} - \frac{1}{2N} \frac{F''(u^{P,N}_{k}(t))}{F'(u^{P,N}_{k}(t))^{2}} \Biggl\vert -u^{P,N}_{k}(t) + \sum_{j=1}^{P} w_{kj} F \bigl(u^{P,N}_{j}(t)\bigr) \Biggr\vert \Biggr) \,dt \\ & {} + \frac{1}{\sqrt{N}} \biggl(\frac{ |-u^{P,N}_{k}(t) + \sum_{j=1}^{P} w_{kj} F(u^{P,N}_{j}(t)) |}{F'(u^{P,N}_{k}(t))} \biggr)^{\frac {1}{2}} \,dB_{k}(t). \end{aligned} \end{aligned}$$

Since the square root function is not Lipschitz continuous near 0, we cannot apply standard existence theorems to obtain a solution to (13) with the full multiplicative noise term. Instead we will linearize around a deterministic solution to the neural field equation and approximate to a certain order of $\frac{1}{\sqrt{N}}$.

### Fluctuations around the Traveling Wave

Let *ū* be a solution to the neural field equation (1). To determine the finite-size effects on *ū*, we consider a spatially extended network, that is, we look at populations distributed over an interval $[-L,L] \subset \mathbb {R}$ and use the stochastic integral derived in Proposition 2 to describe the local fluctuations on this interval.

Let $m \in\mathbb{N}$ be the density of populations on $[-L,L]$ and consider $P=2mL$ populations located at $\frac{k}{m}$, $k \in\{-mL, -mL+1, \ldots, mL-1\}$. We choose the weights $w_{kl}$ as a discretization of the integral kernel $w:\mathbb {R}\rightarrow[0,\infty)$,

14 $$ w^{m}_{kl} = \int_{\frac{l}{m}}^{\frac{l+1}{m}} w\biggl(\frac{k}{m}- y\biggr) \,dy,\quad -mL \leq k,l \leq mL-1. $$

Since we think of the network as describing only a section of the actual domain $\mathbb {R}$, we add to each population an input $F(\bar {u}_{t}(-L))$ and $F(\bar{u}_{t}(L))$, respectively, at the boundaries with corresponding weights

15 $$\begin{aligned} \begin{aligned} w^{m,+}_{k} & = \int_{L}^{\infty} w\biggl(\frac{k}{m} - y\biggr) \,dy, \\ w^{m,-}_{k} & = \int_{- \infty}^{-L} w\biggl(\frac{k}{m} - y\biggr) \,dy. \end{aligned} \end{aligned}$$

Fix a population size $N \in \mathbb {N}$. Set $\bar{u}_{k}(t) = \bar {u}(\frac{k}{m}, t)$ and for $u\in \mathbb {R}^{P}$,

$$\hat{b}^{m}_{k}(t,u) = - u_{k}(t) + \sum _{l=-mL}^{mL-1} w^{m}_{kl} F \bigl(u_{l}(t)\bigr) + w_{k}^{m,+} F\bigl( \bar{u}(L,t)\bigr) + w_{k}^{m,-} F\bigl(\bar{u}(-L,t)\bigr). $$

We write

16 $$ u_{k} = \bar{u}_{k}+v_{k} $$

and assume that $v_{k}$ is of order $1/\sqrt{N}$. Linearizing (13) around $(\bar{u}_{k})$ we obtain the approximation

$$du_{k}(t) = \hat{b}_{k}^{m}(t,u) \,dt + \frac{1}{\sqrt{N F'(\bar{u}_{k}(t))}} \bigl\vert \hat{b}^{m}_{k}(t,\bar{u}) \bigr\vert ^{\frac{1}{2}} \,dB_{k}(t) $$

to order $1/\sqrt{N}$.

Note that $\hat{b}^{m}_{k}(t,\bar{u}) \approx\partial_{t} \bar{u}_{k}(t) = 0$ for a stationary solution *ū*, with equality if *ū* is constant. The finite-size effects are hence of smaller order. Since the square root function is not differentiable at 0 we cannot expand further.

However, the situation is different if we linearize around a moving pattern. We consider the traveling wave solution $u^{\mathrm{TW}}_{t}(x) = \hat {u}(x-ct)$ to (1) and we assume without loss of generality that $c>0$. Then $\hat{b}^{m}_{k}(t,\bar{u}) \approx\partial_{t} \bar {u}_{k}(t) = - c \partial_{x} u^{\mathrm{TW}}_{t} <0$. This monotonicity property allows us to approximate to order $1/N$ in (13). Indeed, note that since *û* and *F* are increasing,

17 $$\begin{aligned} & {-}\hat{b}^{m}_{k}\bigl(t, u^{\mathrm{TW}}_{t}\bigr) \\ &\quad = u^{\mathrm{TW}}_{t}\biggl(\frac{k}{m}\biggr) - \sum _{l} w^{m}_{kl} F \biggl(u^{\mathrm{TW}}_{t}\biggl(\frac{l}{m}\biggr)\biggr) \\ &\qquad {}- w_{k}^{m,+} F\bigl(u^{\mathrm{TW}}_{t}(L)\bigr) - w_{k}^{m,-} F\bigl(u^{\mathrm{TW}}_{t}(-L)\bigr) \\ &\quad \geq u^{\mathrm{TW}}_{t}\biggl(\frac{k}{m}\biggr) - \int_{-L}^{\infty} w\biggl(\frac{k}{m}-y\biggr) F \bigl(u^{\mathrm{TW}} _{t}\bigr) (y) \,dy \\ &\qquad {}- \int_{-\infty}^{-L} w\biggl(\frac{k}{m}-y\biggr) F\bigl(u^{\mathrm{TW}}_{t}(-L)\bigr)\,dy \\ &\quad = c\hat{u}_{x}\biggl(\frac{k}{m}-ct\biggr) - \int_{-\infty}^{-L} w\biggl(\frac{k}{m}-y\biggr) \bigl( F\bigl(u^{\mathrm{TW}}_{t}(-L)\bigr)-F\bigl(u^{\mathrm{TW}}_{t}(y) \bigr)\bigr) \,dy \\ &\quad \geq c\hat{u}_{x}\biggl(\frac{k}{m}-ct\biggr) - \bigl( F\bigl(u^{\mathrm{TW}}_{t}(-L)\bigr) - F(a_{1}) \bigr) \\ &\quad \xrightarrow{L\rightarrow\infty} c\hat{u}_{x}\biggl( \frac{k}{m}-ct\biggr) > 0. \end{aligned}$$

So for *L* large enough, $-\hat{b}^{m}_{k}(t, u^{\mathrm{TW}}_{t})>0$ and we have, using Taylor’s formula and (16),

$$\begin{aligned} \biggl(\frac{|\hat{b}^{m}_{k}(t,u)|}{F'(u_{k}(t))} \biggr)^{\frac{1}{2}} =& \biggl(\frac{-\hat{b}^{m}_{k}(t, u^{\mathrm{TW}}_{t})}{F'(u^{\mathrm{TW}}_{t}(\frac{k}{m}))} \biggr)^{\frac{1}{2}} + \frac{1}{2\sqrt{-\hat{b}^{m}_{k}(t, u^{\mathrm{TW}}_{t}) F'(u^{\mathrm{TW}}_{t}(\frac{k}{m}))}} \\ &{}\times \biggl( \frac{F''(u^{\mathrm{TW}}_{t}(\frac{k}{m}))}{F'(u^{\mathrm{TW}}_{t}(\frac{k}{m}))} \hat {b}^{m}_{k} \bigl(t, u^{\mathrm{TW}}_{t}\bigr) v_{k}(t) \\ & {} + v_{k}(t) - \sum_{l} w_{kl} F'\biggl(u^{\mathrm{TW}}_{t}\biggl( \frac{l}{m}\biggr)\biggr) v_{l}(t) \biggr) + O \biggl( \frac{1}{N} \biggr). \end{aligned}$$

As a possible diffusion approximation in the case of traveling wave solutions we therefore obtain the system of stochastic differential equations

18 $$\begin{aligned} du_{k}(t) =& \biggl(\hat{b}^{m}_{k}(t, u) + \frac{1}{2N} \frac{F''(u^{\mathrm{TW}}_{t}(\frac {k}{m}))}{F'(u^{\mathrm{TW}}_{t}(\frac{k}{m}))^{2}} \hat{b}^{m}_{k}\bigl(t, u^{\mathrm{TW}}_{t}\bigr) \biggr) \,dt \\ & {} + \frac{1}{\sqrt{N}} \biggl[ \biggl( \frac{-\hat{b}^{m}_{k}(t, u^{\mathrm{TW}} _{t})}{F'(u^{\mathrm{TW}}_{t}(\frac{k}{m}))} \biggr)^{\frac{1}{2}} + \frac{1}{2\sqrt {-\hat{b}^{m}_{k}(t, u^{\mathrm{TW}}_{t}) F'(u^{\mathrm{TW}}_{t}(\frac{k}{m}))}} \\ &{}\times \biggl( \frac{F''(u^{\mathrm{TW}}_{t}(\frac{k}{m}))}{F'(u^{\mathrm{TW}}_{t}(\frac {k}{m}))} \hat{b}^{m}_{k} \bigl(t, u^{\mathrm{TW}}_{t}\bigr) v_{k}(t) \\ & {} + v_{k}(t) - \sum_{l} w_{kl} F'\biggl(u^{\mathrm{TW}}_{t}\biggl( \frac{l}{m}\biggr)\biggr) v_{l}(t) \biggr) \biggr] \,dB_{k}(t), \end{aligned}$$

for which there exists a unique solution as we will see in the next section.

## The Continuum Limit

In this section we take the continuum limit of the network of diffusions (18), that is, we let the size of the domain and the density of populations go to infinity in order to obtain a stochastic neural field equation with a noise term describing the fluctuations around the deterministic traveling wave solution due to finite-size effects.

We thus have to deal with functions that ‘look almost like the wave’ and choose to work in the space $\mathcal{S}:= \{ u:\mathbb {R}\rightarrow \mathbb {R}: u - \hat {u}\in L^{2} \}$. Note that since for $u_{1}, u_{2} \in\mathcal{S}$, $\|u_{1}-u_{2}\| < \infty$, the $L^{2}$-norm induces a topology on $\mathcal{S}$.

### A Word on Correlations

Recall the definition of the Markov chain introduced in Sect. 3. Note that as long as we allow only single jumps in the evolution, meaning that there will not be any jumps in the activity in two populations at the same time, the martingales associated with any two populations will be uncorrelated, yielding independent driving Brownian motions in the diffusion limit (cf. Proposition 2).

This only makes sense for populations that are clearly distinguishable. In order to determine the fluctuations around traveling wave solutions, we consider spatially extended networks of populations. The population located at $x \in \mathbb {R}$ is to be understood as the ensemble of all neurons in the *ε*-neighborhood $(x-\varepsilon , x+\varepsilon )$ of *x* for some $\varepsilon > 0$. If we consider two populations located at $x,y \in \mathbb {R}$ with $|x-y| < 2\varepsilon $, then they will overlap. Consequently, simultaneous jumps will occur, leading to correlations between the driving Brownian motions.

Thus the Markov chain model (and the associated diffusion approximation) is only appropriate as long as the distance between the individual populations is large enough. When taking the continuum limit, we therefore adapt the model by introducing correlations between the driving Brownian motions of populations lying close together.

### The Stochastic Neural Field Equation

We start by defining the limiting object. For $u \in\mathcal{S}$ and $t \in[0,T]$ set

$$b(t,u) (x) = - u(x) + \int_{-\infty}^{\infty} w(x-y) F\bigl(u(y)\bigr) \,dy = -u(x) + w \ast F(u) (x) . $$

Let $\mathcal{W}^{Q}$ be a (cylindrical) *Q*-Wiener process on $L^{2}$ with covariance operator $\sqrt{Q}$ given as $\sqrt{Q}h(x) = \int_{-\infty}^{\infty} q(x,y) h(y) \,dy$ for some symmetric kernel $q(x,y)$ with $q(x,\cdot) \in L^{2} \cap L^{1}$ for all $x \in \mathbb {R}$ and $\sup_{x \in \mathbb {R}} (\|q(x,\cdot)\| + \|q(x,\cdot)\|_{1}) < \infty$. (Details on the theory of *Q*-Wiener processes can be found in [23, 24].) We assume that the dispersion coefficient is given as the multiplication operator associated with $\sigma: [0,T] \times\mathcal{S} \rightarrow L^{2}(\mathbb {R})$, which we also denote by *σ*, where *σ* is Lipschitz continuous with respect to the second variable uniformly in $t\leq T$, that is, we assume that there exists $L_{\sigma}>0$ such that, for all $u_{1}, u_{2} \in\mathcal {S}$ and $t \in[0,T]$,

19 $$ \bigl\Vert \sigma(t,u_{1})-\sigma(t,u_{2}) \bigr\Vert \leq L_{\sigma} \Vert u_{1}-u_{2} \Vert . $$

The correlations are described by the kernel *q*. For *f*, *g* in $L^{2}(\mathbb {R})$,

$$\begin{aligned} E\bigl(\bigl\langle f, \mathcal{W}^{Q}_{t} \bigr\rangle \bigl\langle g, \mathcal{W}^{Q}_{t} \bigr\rangle \bigr) &= \int f(x) Qg(x) \,dx \\ &= \int f(x) \int q(x,z) \int q(z,y) g(y) \,dy \,dz \,dx, \end{aligned}$$

so formally,

$$\mbox{`}E\bigl(\mathcal{W}^{Q}_{t}(x) \mathcal{W}^{Q}_{t}(y) \bigr) = E\bigl(\bigl\langle \delta_{x}, \mathcal {W}^{Q}_{t} \bigr\rangle \bigl\langle \delta_{y}, \mathcal{W}^{Q}_{t} \bigr\rangle \bigr) = q \ast q(x,y)\mbox{'}, $$

where we denote by $q\ast q(x,y) $ the integral $\int q(x,z) q(z,y) \,dz$. We could for example take

20 $$q(x,y)=q(x-y)=\frac{1}{2\varepsilon }1_{\left(-\varepsilon ,\varepsilon \right)}(x-y)$$

for some small $\varepsilon > 0$ (cf. Sect. 5.1).

The definition of the stochastic integral $\int_{0}^{t} \sigma(s, u_{s})\, d\mathcal{W}^{Q}_{s}(x)$ w.r.t. the *Q*-Wiener process $\mathcal{W}^{Q}$ requires in particular that the operator $\sigma(t,v) \circ Q^{\frac{1}{2}}$ is Hilbert–Schmidt. In the following, let $L_{2}^{0}$ be the space of all linear operators *L* on $L^{2}$ for which $L\circ Q^{\frac{1}{2}}$ is Hilbert–Schmidt and denote with $\|L\|_{L_{2}^{0}}$ the Hilbert–Schmidt norm of $L\circ Q^{\frac{1}{2}}$.

With the above assumptions on *Q* and *σ* it then follows that $\sigma(t,v) \in L_{2}^{0}$ since by Parseval’s identity

$$\begin{aligned} \bigl\Vert \sigma(t,v) \bigr\Vert _{L_{2}^{0}}^{2} & = \sum _{k} \bigl\Vert \bar{\sigma}(t,v) Q^{\frac{1}{2}} e_{k} \bigr\Vert _{2}^{2} \\ &= \int\sigma (t,v)^{2}(x) \bigl\Vert q(x,\cdot) \bigr\Vert ^{2} \,dx \\ & \leq\sup_{x} \bigl\Vert q(x,\cdot) \bigr\Vert ^{2} \bigl\Vert \sigma(t,v) \bigr\Vert ^{2} < \infty. \end{aligned}$$

Note that, for uncorrelated noise (i.e. $Q=E$), this is not the case. Therefore, in [13] Riedler and Buckwar derive the central limit theorem in the Sobolev space $H^{-\alpha}$. Splitting up the limiting procedures, $N \rightarrow\infty$ and continuum limit, allows us to incorporate correlations and finally to work in the more natural function space $L^{2}$.

#### Proposition 3

*For any initial condition* $u^{0} \in\mathcal{S}$, *the stochastic evolution equation*

21 $$\begin{aligned} \begin{aligned} du_{t}( x) ={}& \biggl( -u_{t} + w \ast F(u_{t}) + \frac{1}{2N} \frac{F''(u^{\mathrm{TW}} _{t})}{F'(u^{\mathrm{TW}}_{t})^{2}} \partial_{t} u^{\mathrm{TW}}_{t} \biggr) \,dt \\ &{}+ \sigma(t, u_{t}) \,d\mathcal{W}^{Q}_{t}(x), \\ u_{0} ={}& u^{0}, \end{aligned} \end{aligned}$$

*has a unique strong* $\mathcal{S}$-*valued solution*. *u* *has a continuous modification*. *For any* $p \geq2$,

$$E \Bigl(\sup_{t\in[0,T]} \bigl\Vert u_{t}-u^{\mathrm{TW}}_{t} \bigr\Vert ^{p} \Bigr) < \infty. $$

For a proof see for example Prop. 6.5.1 in [18].

### Embedding of the Diffusion Processes

As a next step we embed the systems of coupled diffusion processes (18) into $L^{2}(\mathbb {R})$. Let $m \in\mathbb{N}$ be the population density and $L^{m} \in\mathbb {N}$ the length of the domain with $L^{m} \uparrow\infty$ as $m \rightarrow\infty$. For $k \in\{-mL^{m}, -mL^{m}+1, \ldots, mL^{m}-1\}$ set $I^{m}_{k} = [\frac{k}{m}, \frac{k+1}{m})$ and $J^{m}_{k} = (\frac{k}{m}-\frac{1}{4m}, \frac {k}{m}+\frac{1}{4m})$, and let

$$W_{k}^{m}(t)=2m\langle W_{t}^{Q},1_{J_{k}^{m}}\rangle$$

be the average of $\mathcal{W}^{Q}_{t}$ on the interval $J^{m}_{k}$. Then the $W^{m}_{k}$ are one-dimensional Brownian motions with covariances

$$E\left(W_{k}^{m}W_{l}^{m}\right)=4m^{2}\langle \sqrt{Q}1_{J_{k}^{m}},\sqrt{Q}1_{J_{l}^{m}}\rangle =4m^{2}\int_{J_{k}^{m}}\int_{J_{l}^{m}}q*q(y,z)\;dy\;dz.$$

Note that the Brownian motions are independent as long as $m < \frac{1}{4\varepsilon }$.

For $m \in\mathbb{N}$ let $\hat{\sigma}^{m}:[0,T] \times \mathbb {R}^{P} \rightarrow \mathbb {R}^{P}$ and assume that there exists $L_{\hat{\sigma}^{m}} > 0$ such that, for any $t \in[0,T]$ and $u_{1}, u_{2} \in \mathbb {R}^{P}$,

$$\bigl\Vert \hat{\sigma}^{m}(t,u_{1}) - \hat{ \sigma}^{m}(t,u_{2}) \bigr\Vert _{2} \leq L_{\hat{\sigma }^{m}} \Vert u_{1}-u_{2} \Vert _{2}. $$

Consider the system of coupled stochastic differential equations

$$\begin{aligned} du^{m}_{k}(t) = &\biggl[ \hat{b}^{m}_{k} \bigl(t, \bigl(u^{m}_{k}\bigr)\bigr) + \frac{1}{2N} \frac{F''(u^{\mathrm{TW}}_{t}(\frac {k}{m}))}{F'(u^{\mathrm{TW}}_{t}(\frac{k}{m}))^{2}} \hat{b}^{m}_{k}\biggl(t, \biggl(u^{\mathrm{TW}}_{t}\biggl(\frac {k}{m}\biggr) \biggr)\biggr) \\ & {} + w^{m,+}_{k} F\bigl(u^{\mathrm{TW}}_{t} \bigl(L^{m}\bigr)\bigr) + w^{m,-}_{k} F \bigl(u^{\mathrm{TW}}_{t}\bigl(-L^{m}\bigr)\bigr) \biggr] \,dt \\ & {} + \hat{\sigma}_{k}^{m}\bigl(t,u^{m}(t) \bigr) \,dW_{k}^{m}(t),\quad -mL^{m} \leq k \leq mL^{m}-1, \end{aligned}$$

with weights as in (14) and (15).

We identify $u = (u_{k})_{-mL^{m} \leq k \leq mL^{m}-1} \in\mathbb{R}^{P}$ with its piecewise constant interpolation as an element of $L^{2}$ via the embedding

$$\iota^{m}(u)=\sum_{k=-mL^{m}}^{mL^{m}-1}u_{k}1_{I_{k}^{m}}.$$

For $u \in\mathcal{C}(\mathbb{R})$ set

$$\pi^{m}(u)=\sum_{k=-mL^{m}}^{mL^{m}-1}u\left(\frac{k}{m}\right)1_{I_{k}^{m}}.$$

Then $u^{m}_{t} := \iota^{m}((u^{m}_{k}(t))_{k})$ satisfies

22 $$\begin{aligned} du^{m}_{t}(x) =& b^{m}\bigl(t, u^{m}_{t}\bigr) (x) \,dt \\ &{}+ \frac{1}{2N} \pi^{m} \biggl(\frac{F''(u^{\mathrm{TW}} _{t})}{F'(u^{\mathrm{TW}}_{t})^{2}} \biggr) b^{m}\bigl(t, \pi^{m}\bigl(u^{\mathrm{TW}}_{t}\bigr)\bigr) \,dt \\ &{} + \sigma^{m}\bigl(t,u^{m}_{t}\bigr) \circ \varPhi^{m} \,d\mathcal{W}^{Q}_{t}(x), \end{aligned}$$

where $b^{m}:[0,T]\times L^{2}(\mathbb {R}) \rightarrow L^{2}(\mathbb {R})$ and $\varPhi^{m}:L^{2}(\mathbb {R}) \rightarrow L^{2}(\mathbb {R})$ are given as

$$\begin{array}{rcl} b^{m}(t,u) & = & -u_{t}+\sum_{k}[\int_{-L^{m}}^{L^{m}}w\left(\frac{k}{m}-y\right)F(u_{t})(y)\;dy\\ & & +w_{k}^{m,+}F\left(u_{t}^{\mathrm{TW}}\left(L^{m}\right)\right)+w_{k}^{m,-}F\left(u_{t}^{\mathrm{TW}}\left(-L^{m}\right)\right)]1_{I_{k}^{m}},\\ \Phi^{m}(u) & = & 2m\sum_{k=-mL^{m}}^{mL^{m}-1}\langle u,1_{J_{k}^{m}}\rangle 1_{I_{k}^{m}}, \end{array}$$

and where $\sigma^{m}:[0,T] \times L^{2}(\mathbb {R}) \rightarrow L^{2}(\mathbb {R})$ is such that, for $u\in \mathbb {R}^{P}$, $\sigma^{m}(t,\iota^{m}(u))= \hat{\sigma}^{m}_{k}(t,u)$ on $I^{m}_{k}$. We assume joint continuity and Lipschitz continuity in the second variable uniformly in *m* and $t\leq T$, that is, there exists $L_{\sigma}>0$ such that for $u_{1},u_{2} \in L^{2}(\mathbb {R})$ and $t\leq T$,

$$\bigl\Vert \sigma^{m}(t,u_{1})-\sigma^{m}(t,u_{2}) \bigr\Vert \leq L_{\sigma} \Vert u_{1}-u_{2} \Vert . $$

#### Proposition 4

*For any initial condition* $u^{0} \in L^{2}(\mathbb {R})$ *there exists a unique strong* $L^{2}$-*valued solution* $u^{m}$ *to* (22). $u^{m}$ *admits a continuous modification*. *For any* $p\geq2$,

$$E \Bigl(\sup_{t\leq T} \bigl\Vert u^{m}_{t} \bigr\Vert ^{p} \Bigr) < \infty. $$

#### Proof

Again we check that the drift and diffusion coefficients are Lipschitz continuous. Note that

23 $$\begin{aligned} \begin{aligned}[b] \sum_{k} \frac{1}{m} w\biggl( \frac{k}{m}, y\biggr) ={}& \int_{-L^{m}}^{L^{m}} w(x-y) \,dx + \sum _{k} \int_{\frac{k}{m}}^{\frac{k+1}{m}} w\biggl(\frac{k}{m}-y \biggr)-w(x-y) \,dx \\ \leq{}&\|w\|_{1} + \sum_{k} \int_{\frac{k}{m}}^{\frac{k+1}{m}} \int_{\frac {k}{m}}^{\frac{k+1}{m}} \bigl\vert w_{x}(z-y) \bigr\vert \,dz \,dx \\ ={}& 1 + \sum_{k} \frac{1}{m} \int_{\frac{k}{m}}^{\frac{k+1}{m}} \bigl\vert w_{x}(z-y) \bigr\vert \,dz \\ \leq{}&1 + \frac{1}{m}\|w_{x} \|_{1}. \end{aligned} \end{aligned}$$

Therefore, for $u_{1}, u_{2} \in L^{2}(\mathbb {R})$,

$$\begin{aligned} &\bigl\Vert b^{m}(t, u_{1}) - b^{m}(t, u_{2}) \bigr\Vert _{2}^{2} \\ &\quad \leq2 \Vert u_{1}-u_{2} \Vert ^{2} + 2 \bigl\Vert F' \bigr\Vert _{\infty}^{2} \int_{-L^{m}}^{L^{m}} \sum_{k} \frac{1}{m}w\biggl(\frac{k}{m}-y\biggr) \bigl( u_{1}(y) -u_{2}(y) \bigr)^{2} \,dy \\ &\quad \leq2 \Vert u_{1}-u_{2} \Vert ^{2} + 2 \bigl\Vert F' \bigr\Vert _{\infty}^{2} \biggl( 1 + \frac{1}{m} \Vert w_{x} \Vert _{1} \biggr) \Vert u_{1}-u_{2} \Vert ^{2} \end{aligned}$$

and for an orthonormal basis $(e_{k})$ of $L^{2}(\mathbb {R})$ we obtain, using Parseval’s identity,

$$\begin{array}{rl} & {∥\left(\sigma^{m}(t,u_{1})-\sigma^{m}(t,u_{2})\right)\circ \Phi^{m}∥}_{L_{2}^{0}}^{2}\\ & \;=\sum_{k}{∥\left(\sigma^{m}(t,u_{1})-\sigma^{m}(t,u_{2})\right)\sum_{l}2m\langle e_{k},\sqrt{Q}1_{J_{l}^{m}}\rangle 1_{I_{l}^{m}}∥}^{2}\\ & \;=\int {\left(\sigma^{m}(t,u_{1})-\sigma^{m}(t,u_{2})\right)}^{2}(x)\sum_{l}4m^{2}\int {\left(\int_{J_{l}^{m}}q(z,y)\;dy\right)}^{2}\;dz\;1_{I_{l}^{m}}(x)\;dx\\ & \;\leq \int {\left(\sigma^{m}(t,u_{1})-\sigma^{m}(t,u_{2})\right)}^{2}(x)\sum_{l}2m\int \int_{J_{l}^{m}}q{\left(z,y\right)}^{2}\;dy\;dz\;1_{I_{l}^{m}}(x)\;dx\\ & \;=\int {\left(\sigma^{m}(t,u_{1})-\sigma^{m}(t,u_{2})\right)}^{2}(x)\sum_{l}2m\int_{J_{l}^{m}}{∥q(y,\cdot )∥}^{2}\;dy\;1_{I_{l}^{m}}(x)\;dx\\ & \;\leq \underset{x}{\sup}{∥q(x,\cdot )∥}^{2}L_{\sigma }^{2}{∥u_{1}-u_{2}∥}^{2}. \end{array}$$

 □

### Convergence

We are now able to state the main convergence result. We will need the following assumption on the kernel *w*.

#### Assumption 5

There exists $C_{w}>0$ such that, for $x\geq0$,

24 $$ \int_{x}^{\infty} w(y) \,dy \leq C_{w} w(x). $$

That assumption is satisfied for classical choices of *w* such as ${w(x) = \frac{1}{2\sigma}e^{-\frac{|x|}{\sigma}}}$ or ${w(x) = \frac {1}{\sqrt{2\pi\sigma^{2}}} e^{-\frac{x^{2}}{2\sigma^{2}}}}$.

#### Theorem 6

*Fix* $T>0$. *Let* *u* *and* $u^{m}$ *be the solutions to* (21) *and* (22), *respectively*. *Assume that*

(i) $\sup_{k}\sup_{x\in I_{k}^{m}}∥2m\sqrt{Q}1_{J_{k}^{m}}-q(x,\cdot )∥\overset{m\to \infty }{\to }0$,
(ii) *for any* $u: [0,T] \rightarrow\mathcal{S}$ *with* $\sup_{t\leq T} \| u_{t} - \hat {u}\| < \infty$,
  $$\underset{t\leq T}{\sup}∥\sigma^{m}(t,u_{t}1_{\left(-L^{m},L^{m}\right)})-\sigma (t,u_{t})∥\overset{m\to \infty }{\to }0.$$

*Then*, *for any initial conditions* ${u_{0}^{m}\in L^{2}(\mathbb {R})}$, ${u_{0} \in\mathcal{S}}$ *such that*

$$\bigl\| u_{0}^{m}-u_{0}\bigr\| _{L^{2}((-L^{m}, L^{m}))} \xrightarrow{m\rightarrow\infty} 0, $$

*and for all* $p\geq2$,

$$\mathbb{E} \Bigl( \sup_{t \in[0,T]} \bigl\Vert u^{m}_{t}-u_{t} \bigr\Vert _{L^{2}((-L^{m}, L^{m}))}^{p} \Bigr) \xrightarrow{m \rightarrow\infty} 0. $$

We postpone the proof to the Appendix.

#### Remark 7

Let $\varepsilon > 0$. The kernel $q(x,y)=\frac{1}{2\varepsilon }1_{\left(x-\varepsilon ,x+\varepsilon \right)}(y)$ satisfies assumption (i) of the theorem. Indeed, note that, for *x*, *z* with $|x-z| \leq\frac{1}{m}$, $|\{y:1_{\left(x-\varepsilon ,x+\varepsilon \right)}(y)\neq 1_{\left(z-\varepsilon ,z+\varepsilon \right)}(y)\}|\leq |z-x|\leq \frac{1}{m}$. Therefore we obtain, for all *k* and for any $x \in I^{m}_{k}$,

$$\begin{array}{rl} {∥2m\sqrt{Q}1_{J_{k}^{m}}-q(x,\cdot )∥}_{2}^{2} & =4m^{2}\int_{-\infty }^{\infty }{\left(\int_{J_{k}^{m}}q(z,y)-q(x,y)\;dz\right)}^{2}\;dy\\ & \leq 2m\int_{-\infty }^{\infty }\int_{J_{k}^{m}}{\left(q(z,y)-q(x,y)\right)}^{2}\;dz\;dy\\ & \leq 2\int_{J_{k}^{m}}\frac{1}{4\varepsilon^{2}}\;dz\leq \frac{1}{4\varepsilon^{2}m}\overset{m\to \infty }{\to }0. \end{array}$$

Theorem 6 can now be applied to the asymptotic expansion of the fluctuations around the traveling wave derived in Sect. 4.2. In order to ensure that the diffusion coefficients are in $L^{2}(\mathbb {R})$, we cut off the noise outside a compact set $\{ \partial_{x} u^{\mathrm{TW}}_{t} \geq\delta\}$, $\delta>0$. Note that the neglected region moves with the wave such that we always retain the fluctuations in the relevant regime away from the fixed points.

#### Theorem 8

*Assume that the wave speed is strictly positive*, $c>0$. *Fix* $\delta>0$. *The diffusion coefficients as derived in Sect*. 4.2,

$$\begin{array}{rcl} \sigma (t,u) & = & \frac{1}{\sqrt{N}}(\alpha (t)+\beta (t)\left(u-u_{t}^{\mathrm{TW}}\right)\\ & & -\gamma (t)w*\left(F^{\prime }\left(u_{t}^{\mathrm{TW}}\right)\left(u-u_{t}^{\mathrm{TW}}\right)\right))1_{\left\{\partial_{x}u_{t}^{\mathrm{TW}}\geq \delta \right\}},\\ \sigma^{m}(t,u) & = & \frac{1}{\sqrt{N}}(\alpha^{m}(t)+\beta^{m}(t)\left(u-\pi^{m}\left(u_{t}^{\mathrm{TW}}\right)\right)\\ & & -\gamma^{m}(t)\pi^{m}\left(w*\left(\pi^{m}\left(F^{\prime }\left(u_{t}^{\mathrm{TW}}\right)\right)\left(u-\pi^{m}\left(u_{t}^{\mathrm{TW}}\right)\right)\right)\right))1_{\left\{\partial_{x}u_{t}^{\mathrm{TW}}\geq \delta \right\}}, \end{array}$$

*where*

$$\begin{array}{rcl} \alpha (t) & = & \sqrt{\frac{\left|-u_{t}^{\mathrm{TW}}+w*F(u_{t}^{\mathrm{TW}})\right|}{F^{\prime }(u_{t}^{\mathrm{TW}})}}=\sqrt{\frac{c\partial_{x}u_{t}^{\mathrm{TW}}}{F^{\prime }(u_{t}^{\mathrm{TW}})}},\\ \beta (t) & = & \frac{1}{2\sqrt{c\partial_{x}u_{t}^{\mathrm{TW}}F^{\prime }(u_{t}^{\mathrm{TW}})}}\left(-\frac{F^{\prime \prime }(u_{t}^{\mathrm{TW}}(x))}{F^{\prime }(u_{t}^{\mathrm{TW}}(x))}c\partial_{x}u_{t}^{\mathrm{TW}}+1\right),\\ \gamma (t) & = & \frac{1}{2\sqrt{c\partial_{x}u_{t}^{\mathrm{TW}}F^{\prime }(u_{t}^{\mathrm{TW}})}},\\ \alpha^{m}(t) & = & \sqrt{\frac{-b^{m}(t,\pi^{m}(u_{t}^{\mathrm{TW}}))}{\pi^{m}(F^{\prime }(u_{t}^{\mathrm{TW}}))}}1_{\left[-L^{m},L^{m}\right)},\\ \beta^{m}(t) & = & \frac{1}{2\sqrt{-b^{m}(t,\pi^{m}(u_{t}^{\mathrm{TW}}))\pi^{m}(F^{\prime }(u_{t}^{\mathrm{TW}}))}}\\ & & \times \left(-\frac{\pi^{m}(F^{\prime \prime }(u_{t}^{\mathrm{TW}}))}{\pi^{m}(F^{\prime }(u_{t}^{\mathrm{TW}}))}\left(-b^{m}\left(t,\pi^{m}\left(u_{t}^{\mathrm{TW}}\right)\right)\right)+1\right)1_{\left[-L^{m},L^{m}\right)},\\ \gamma^{m}(t) & = & \frac{1}{2\sqrt{-b^{m}(t,\pi^{m}(u_{t}^{\mathrm{TW}}))\pi^{m}(F^{\prime }(u_{t}^{\mathrm{TW}}))}}1_{\left[-L^{m},L^{m}\right)}, \end{array}$$

*are jointly continuous and Lipschitz continuous in the second variable with Lipschitz constant uniform in* *m* *and* $t\leq T$, *and satisfy condition* (ii) *of Theorem* 6.

For a proof see Thm. 6.5.5 in [18].

## Summary and Conclusions

In this paper we have investigated the derivation of neural field equations from detailed neuron models. Following a common approach in the literature, we started from a phenomenological Markov chain model that yields the neural field equation in the infinite-population limit and allows one to obtain stochastic or deterministic corrections for networks of finite populations. As one novelty to the existing literature we considered a new choice of jump rates, given in the activity-based setting as

25 $$ q^{P,N} \biggl( x, x\pm\frac{1}{N}e_{i} \biggr) = N \Biggl( - x_{i} + F \Biggl( \sum _{j=1}^{P} w_{ij} x_{j} \Biggr) \Biggr)_{\pm} , $$

and showed that it leads to qualitatively different results in dynamical states with high (resp. low) activity in comparison with the rates

26 $$\begin{aligned} \begin{aligned} \tilde{q}\biggl(x, x+ \frac{1}{N}e_{i}\biggr) & = N F \Biggl( \sum _{j=1}^{P} w_{ij} x_{j} \Biggr) , \\ \tilde{q}\biggl(x, x-\frac{1}{N}e_{i}\biggr) & = N x_{i} , \end{aligned} \end{aligned}$$

considered in the literature so far (see for example [11, 13]).

The following two major reasons make us believe that the rates (25) provide a better description of finite-size effects in neural fields.

1. The neural field equations evolve on a different time scale than single neuron activity. In the activity-based setting they describe a coarse-grained time-averaged population activity and in the voltage-based setting they are derived under the assumption that the activity in each population is slowly varying such that one can assume that it is related to the population potential via $a = F (u)$. The rates (25) reflect this property more than the rates (26), in particular in regimes with high (resp. low) activity.
2. Fluctuations due to finite-size effects should be strong where $F^{\prime}\gg1$, and therefore small changes in the synaptic input lead to comparably large changes in the population activity. They should become small, however, where $F^{\prime}\ll1$, so in particular around the stable fixed points of the system. This is captured in the jump rates (25), whereas in case (26), fluctuations are high where the activity is high.

The corrections to the neural field equation derived in this setting are of a qualitatively different form than the ones previously considered. As a consequence, one should reconsider effects of intrinsic noise due to finite-size effects as studied for example in [25]. In particular the lowest order correction to the moment equations, or the additive part of the stochastic correction, vanishes when linearizing around a stationary solution. The noise is therefore of smaller order than previously assumed.

This is not the case for stable moving patterns like traveling fronts or pulses, suggesting the movement as a main source of noise. We have for the first time rigorously derived a well-posed stochastic continuum neural field equation with an additive noise term that can be used to study the finite-size effects on these kinds of solutions. It is particularly suitable for the analysis of traveling wave solutions, since the monotonicity of the solution allows one to consider a multiplicative noise term as derived in Sect. 4.2.

## Appendix: Proof of Theorem 6

Set $v_{t} = u_{t} - u^{\mathrm{TW}}_{t}$ and $v_{t}^{m} = u^{m}_{t} - \pi^{m}(u^{\mathrm{TW}}_{t})$. Note that

27 $$\begin{aligned} &\int_{-L^{m}}^{L^{m}} \bigl(\pi^{m} \bigl(u^{\mathrm{TW}}_{t}\bigr) (x)-u^{\mathrm{TW}}_{t}(x) \bigr)^{2} \,dx \\ &\quad \leq\sum_{k} \int_{\frac{k}{m}}^{\frac{k+1}{m}} \biggl( \int_{\frac {k}{m}}^{\frac{k+1}{m}} \partial_{x} u^{\mathrm{TW}}_{t}(z) \,dz \biggr)^{2} \,dx \\ & \quad \leq\frac{1}{m^{2}} \sum_{k} \int_{\frac{k}{m}}^{\frac{k+1}{m}} \bigl( \partial_{x} u^{\mathrm{TW}}_{t}(z) \bigr)^{2} \,dz \leq \frac{1}{m^{2}} \|\hat {u}_{x}\|^{2}. \end{aligned}$$

For the proof of the theorem it therefore suffices to show that

$$E \Bigl(\sup_{t\leq T} \bigl\Vert v_{t}-v^{m}_{t} \bigr\Vert _{2}^{p} \Bigr) \xrightarrow{m\rightarrow \infty} 0, $$

since this will imply that

$$\begin{aligned} & E \Bigl( \sup_{t\leq T} \bigl\Vert u_{t} - u^{m}_{t} \bigr\Vert _{L^{2}((-L^{m}, L^{m}))}^{p} \Bigr) \\ &\quad \leq \mathit{const} \times \Bigl[E \Bigl(\sup_{t\leq T} \bigl\Vert u^{\mathrm{TW}}_{t}-\pi^{m}\bigl(u^{\mathrm{TW}}_{t} \bigr) \bigr\Vert _{L^{2}((-L^{m}, L^{m}))}^{p} \Bigr) + E \Bigl( \sup _{t\leq T} \bigl\Vert v_{t}-v^{m}_{t} \bigr\Vert _{2}^{p} \Bigr) \Bigr] \\ &\quad \xrightarrow{m \rightarrow\infty} 0. \end{aligned}$$

By Itô’s formula,

$$\begin{aligned} & \frac{1}{2} d \bigl\Vert v^{m}_{t}-v_{t} \bigr\Vert _{2}^{2} \\ &\quad = \bigl\langle b^{m}\bigl(t, v^{m}_{t}+ \pi^{m}\bigl(u^{\mathrm{TW}}_{t}\bigr)\bigr) - b\bigl(t, v_{t}+u^{\mathrm{TW}}_{t}\bigr) - \pi^{m} \bigl( \partial_{t}u^{\mathrm{TW}}_{t}\bigr) + \partial_{t} u^{\mathrm{TW}}_{t}, v^{m}_{t}-v_{t} \bigr\rangle \,dt \\ &\qquad {} + \frac{1}{2N} \biggl\langle \pi^{m} \biggl( \frac{F''(u^{\mathrm{TW}} _{t})}{F'(u^{\mathrm{TW}}_{t})^{2}} \biggr) b^{m}\bigl(t, \pi^{m} \bigl(u^{\mathrm{TW}}_{t}\bigr)\bigr) - \frac{F''(u^{\mathrm{TW}}_{t})}{F'(u^{\mathrm{TW}} _{t})^{2}} \partial_{t} u^{\mathrm{TW}}_{t}, v^{m}_{t}-v_{t} \biggr\rangle \,dt \\ &\qquad {} + \frac{1}{2} \bigl\Vert \sigma^{m} \bigl(t,v^{m}_{t}+ \pi^{m}\bigl(u^{\mathrm{TW}}_{t} \bigr)\bigr) \circ\varPhi ^{m} - \sigma\bigl(t,v_{t}+u^{\mathrm{TW}}_{t} \bigr) \bigr\Vert _{L^{0}_{2}}^{2} \,dt \\ &\qquad {} + \bigl\langle v^{m}_{t}-v_{t}, \bigl( \sigma^{m}\bigl(t, v^{m}_{t}+\pi^{m} \bigl(u^{\mathrm{TW}} _{t}\bigr)\bigr) \circ\varPhi^{m} - \sigma\bigl(t, v_{t}+u^{\mathrm{TW}}_{t}\bigr) \bigr) \,d \mathcal{W}^{Q}_{t} \bigr\rangle . \end{aligned}$$

In order to finally apply Gronwall’s lemma, we estimate the terms one by one.

### A.1 The Drift

We start by regrouping the terms in a suitable way. We have

$$\begin{array}{rl} & {∥v_{t}^{m}-v_{t}+b^{m}\left(t,v_{t}^{m}+\pi^{m}\left(u_{t}^{\mathrm{TW}}\right)\right)-b\left(t,v_{t}+u_{t}^{\mathrm{TW}}\right)-\pi^{m}\left(\partial_{t}u_{t}^{\mathrm{TW}}\right)+\partial_{t}u_{t}^{\mathrm{TW}}∥}^{2}\\ & \;=\int_{-\infty }^{\infty }[\sum_{k}(\int_{-L^{m}}^{L^{m}}w\left(\frac{k}{m}-y\right)F\left(v_{t}^{m}(y)+\pi^{m}\left(u_{t}^{\mathrm{TW}}\right)(y)\right)\;dy\\ & \;+\int_{L^{m}}^{\infty }w\left(\frac{k}{m}-y\right)F\left(u_{t}^{\mathrm{TW}}\left(L^{m}\right)\right)\;dy\\ & \;+\int_{-\infty }^{-L^{m}}w\left(\frac{k}{m}-y\right)F\left(u_{t}^{\mathrm{TW}}\left(-L^{m}\right)\right)\;dy)1_{I_{k}^{m}}(x)\\ & \;-\int_{-\infty }^{\infty }w(x-y)F\left(v_{t}(y)+u_{t}^{\mathrm{TW}}(y)\right)\;dy\\ & \;-\pi^{m}\left(w*F\left(u_{t}^{\mathrm{TW}}\right)\right)+w*F\left(u_{t}^{\mathrm{TW}}\right)(x){]}^{2}\;dx\\ & \;\leq 6\int_{-\infty }^{\infty }[\sum_{k}\int_{-L^{m}}^{L^{m}}w\left(\frac{k}{m}-y\right)(F\left(v_{t}^{m}(y)+\pi^{m}\left(u_{t}^{\mathrm{TW}}\right)(y)\right)\\ & \;-F\left(v_{t}(y)+u_{t}^{\mathrm{TW}}(y)\right))\;dy1_{I_{k}^{m}}(x){]}^{2}\\ & \;+[\sum_{k}\int_{-L^{m}}^{L^{m}}\left(w\left(\frac{k}{m}-y\right)-w(x-y)\right)(F\left(v_{t}(y)+u_{t}^{\mathrm{TW}}(y)\right)\\ & \;-F\left(u_{t}^{\mathrm{TW}}(y)\right))\;dy1_{I_{k}^{m}}(x){]}^{2}\\ & \;+{\left[\sum_{k}\int_{L^{m}}^{\infty }w\left(\frac{k}{m}-y\right)\left(F\left(u_{t}^{\mathrm{TW}}\left(L^{m}\right)\right)-F\left(u_{t}^{\mathrm{TW}}(y)\right)\right)\;dy1_{I_{k}^{m}}(x)\right]}^{2}\\ & \;+{\left[\sum_{k}\int_{-\infty }^{-L^{m}}w\left(\frac{k}{m}-y\right)\left(F\left(u_{t}^{\mathrm{TW}}\left(-L^{m}\right)\right)-F\left(u_{t}^{\mathrm{TW}}(y)\right)\right)\;dy1_{I_{k}^{m}}(x)\right]}^{2}\\ & \;+{\left[\left(\int_{L^{m}}^{\infty }+\int_{-\infty }^{-L^{m}}\right)w(x-y)\left(F\left(v_{t}(y)+u_{t}^{\mathrm{TW}}(y)\right)-F\left(u_{t}^{\mathrm{TW}}(y)\right)\right)\;dy\right]}^{2}\\ & \;+[\int_{-L^{m}}^{L^{m}}w(x-y)(F\left(v_{t}(y)+u_{t}^{\mathrm{TW}}(y)\right)\\ & \;-F\left(u_{t}^{\mathrm{TW}}(y)\right))\;dy1_{\left(-\infty ,-L^{m})\cup [L^{m},\infty \right)}(x){]}^{2}\;dx\\ & \;=:6(S_{1}+S_{2}+S_{3}+S_{4}+S_{5}+S_{6}). \end{array}$$

Using the Cauchy–Schwarz inequality we get

$$\begin{aligned} S_{1} \leq& \bigl\Vert F' \bigr\Vert _{\infty}^{2} \int_{-L^{m}}^{L^{m}} \sum_{k} \frac{1}{m} w\biggl(\frac {k}{m}- y\biggr) \\ &{}\times \bigl( v^{m}_{t}(y) + \pi^{m}\bigl(u^{\mathrm{TW}}_{t} \bigr) (y)- v_{t}(y)-u^{\mathrm{TW}}_{t}(y) \bigr)^{2} \,dy \\ \stackrel{\text{(23)}}{\leq}& 2 \biggl(1 + \frac{1}{m} \Vert w_{x} \Vert _{1} \biggr) \bigl\Vert F' \bigr\Vert _{\infty}^{2} \\ &{}\times\biggl( \bigl\Vert v^{m}_{t}-v_{t} \bigr\Vert _{2}^{2} + \int_{-L^{m}}^{L^{m}} \bigl(\pi^{m} \bigl(u^{\mathrm{TW}}_{t}\bigr) (y)-u^{\mathrm{TW}}_{t}(y) \bigr)^{2} \,dy \biggr). \end{aligned}$$

With (27) it follows that

$$S_{1} \leq2 \biggl(1 + \frac{1}{m} \Vert w_{x} \Vert _{1} \biggr) \bigl\Vert F' \bigr\Vert _{\infty}^{2} \biggl( \bigl\Vert v^{m}_{t}-v_{t} \bigr\Vert _{2}^{2} + \frac{1}{m^{2}} \Vert \hat {u}_{x} \Vert _{2}^{2} \biggr). $$

Another application of the Cauchy–Schwarz inequality yields

$$\begin{aligned} S_{2} =& \sum_{k} \int_{\frac{k}{m}}^{\frac{k+1}{m}} \biggl( \int_{-L^{m}}^{L^{m}} \biggl( w\biggl(\frac{k}{m}- y \biggr) - w(x-y) \biggr) \\ &{}\times \bigl( F\bigl(v_{t}(y)+u^{\mathrm{TW}}_{t}(y) \bigr)-F\bigl(u^{\mathrm{TW}}_{t}(y)\bigr) \bigr) \,dy \biggr)^{2} \,dx \\ \leq&\sum_{k} \frac{1}{m} \biggl( \int_{-L^{m}}^{L^{m}} \int_{\frac {k}{m}}^{\frac{k+1}{m}} \bigl\vert w_{x}(z-y) \bigr\vert \,dz \bigl( F\bigl(v_{t}(y)+u^{\mathrm{TW}}_{t}(y) \bigr)-F\bigl(u^{\mathrm{TW}} _{t}(y)\bigr) \bigr) \,dy \biggr)^{2} \\ \leq&\sum_{k} \frac{1}{m} \int_{-L^{m}}^{L^{m}} \int_{\frac{k}{m}}^{\frac {k+1}{m}} \bigl\vert w_{x}(z-y) \bigr\vert \,dz \,dy \\ & {} \times \int_{-L^{m}}^{L^{m}} \int_{\frac{k}{m}}^{\frac {k+1}{m}} \bigl\vert w_{x}(z-y) \bigr\vert \,dz \bigl( F\bigl(v_{t}(y)+u^{\mathrm{TW}}_{t}(y) \bigr)-F\bigl(u^{\mathrm{TW}}_{t}(y)\bigr) \bigr)^{2} \,dy \\ \leq&\frac{1}{m^{2}} \Vert w_{x} \Vert _{1}^{2} \bigl\Vert F' \bigr\Vert _{\infty}^{2} \Vert v_{t} \Vert _{2}^{2} . \end{aligned}$$

Using integration by parts, (23), and assumption (24), we obtain

$$\begin{aligned} S_{3} =& \sum_{k} \frac{1}{m} \biggl( \underbrace{ \biggl[ - \int_{y}^{\infty} w\biggl(z-\frac{k}{m}\biggr) \,dz \bigl( F\bigl(u^{\mathrm{TW}}_{t}\bigl(L^{m}\bigr)\bigr) - F\bigl(u^{\mathrm{TW}}_{t}(y)\bigr) \bigr) \biggr]_{y=L^{m}}^{\infty}}_{=0} \\ &{} - \int_{L^{m}}^{\infty} \int_{y}^{\infty} w\biggl(z-\frac{k}{m}\biggr) \,dz F'\bigl(u^{\mathrm{TW}}_{t}(y)\bigr) \partial_{x}u^{\mathrm{TW}}_{t}(y) \,dy \biggr)^{2} \\ \leq &C_{w}^{2} \sum_{k} \frac{1}{m} \biggl( \int_{L^{m}}^{\infty} w\biggl(y-\frac {k}{m}\biggr) F'\bigl(u^{\mathrm{TW}}_{t}(y)\bigr) \partial_{x} u^{\mathrm{TW}}_{t}(y) \,dy \biggr)^{2} \\ \leq& C_{w}^{2} \biggl(1 + \frac{1}{m} \Vert w_{x} \Vert _{1} \biggr) \bigl\Vert F' \bigr\Vert _{\infty}^{2} \int _{L^{m}}^{\infty} \bigl(\partial_{x}u^{\mathrm{TW}}_{t}(y) \bigr)^{2} \,dy. \end{aligned}$$

Analogously,

$$S_{4} \leq C_{w}^{2} \biggl(1 + \frac{1}{m} \Vert w_{x} \Vert _{1} \biggr) \bigl\Vert F' \bigr\Vert _{\infty}^{2} \int _{-\infty}^{-L^{m}} \bigl(\partial_{x} u^{\mathrm{TW}}_{t}(y)\bigr)^{2} \,dy. $$

Finally we observe that

$$ S_{5} \leq \bigl\Vert F' \bigr\Vert _{\infty}^{2} \biggl( \int_{L^{m}}^{\infty} + \int_{-\infty }^{-L^{m}} \biggr) v_{t}^{2}(y) \,dy $$

and

$$\begin{aligned} S_{6} & \leq \bigl\Vert F' \bigr\Vert _{\infty}^{2} \biggl( \int_{L^{m}}^{\infty} + \int_{-\infty }^{-L^{m}} \biggr) \biggl( \int_{-L^{m}}^{L^{m}} w(x-y) \bigl\vert v_{t}(y) \bigr\vert \,dy \biggr)^{2} \,dx \\ & \leq \bigl\Vert F' \bigr\Vert _{\infty}^{2} \biggl( \int_{L^{m}}^{\infty} + \int_{-\infty }^{-L^{m}} \biggr) \bigl( w \ast|v_{t}| (x) \bigr)^{2} \,dx. \end{aligned}$$

Finally we consider

$$\begin{array}{rcl} S_{7} & := & {∥\frac{F^{\prime \prime }(u_{t}^{\mathrm{TW}})}{F^{\prime }{\left(u_{t}^{\mathrm{TW}}\right)}^{2}}\partial_{t}u_{t}^{\mathrm{TW}}-\pi^{m}\left(\frac{F^{\prime \prime }(u_{t}^{\mathrm{TW}})}{F^{\prime }{\left(u_{t}^{\mathrm{TW}}\right)}^{2}}\right)b^{m}\left(t,\pi^{m}\left(u_{t}^{\mathrm{TW}}\right)\right)∥}^{2}\\ & \leq & 4[\left(\int_{-\infty }^{-L^{m}}+\int_{L^{m}}^{\infty }\right){\left(\frac{F^{\prime \prime }(u_{t}^{\mathrm{TW}})(y)}{F^{\prime }{\left(u_{t}^{\mathrm{TW}}\right)}^{2}(y)}\partial_{t}u_{t}^{\mathrm{TW}}(y)\right)}^{2}\;dy\\ & & +{∥\left(\frac{F^{\prime \prime }(u_{t}^{\mathrm{TW}})}{F^{\prime }{\left(u_{t}^{\mathrm{TW}}\right)}^{2}}-\pi^{m}\left(\frac{F^{\prime \prime }(u_{t}^{\mathrm{TW}})}{F^{\prime }{\left(u_{t}^{\mathrm{TW}}\right)}^{2}}\right)\right)\partial_{t}u_{t}^{\mathrm{TW}}1_{\left(-L^{m},L^{m}\right)}∥}^{2}\\ & & +{∥\pi^{m}\left(\frac{F^{\prime \prime }(u_{t}^{\mathrm{TW}})}{F^{\prime }{\left(u_{t}^{\mathrm{TW}}\right)}^{2}}\right)\left(\partial_{t}u_{t}^{\mathrm{TW}}-\pi^{m}\left(\partial_{t}u_{t}^{\mathrm{TW}}\right)\right)1_{\left(-L^{m},L^{m}\right)}∥}^{2}\\ & & +∥\pi^{m}\left(\frac{F^{\prime \prime }(u_{t}^{\mathrm{TW}})}{F^{\prime }{\left(u_{t}^{\mathrm{TW}}\right)}^{2}}\right)(\pi^{m}\left(w*F\left(u_{t}^{\mathrm{TW}}\right)\right)-\pi^{m}\left(w*\pi^{m}\left(F\left(u_{t}^{\mathrm{TW}}\right)\right)\right)\\ & & -\sum_{k}\left(w_{k}^{m,+}F\left(u_{t}^{\mathrm{TW}}\left(L^{m}\right)\right)+w_{k}^{m,-}F\left(u_{t}^{\mathrm{TW}}\right)\left(-L^{m}\right)\right)1_{I_{k}^{m}}){∥}^{2}]\\ & = & 4(S_{7,1}+S_{7,2}+S_{7,3}+S_{7,4}). \end{array}$$

We have

$$ S_{7,2} \leq \biggl\Vert \frac{F^{(3)}(\hat {u}) \hat {u}_{x}}{(F'(\hat {u}))^{2}} - \frac{2 (F''(\hat {u}))^{2}\hat {u}_{x}}{(F'(\hat {u}))^{3}} \biggr\Vert _{\infty}^{2} \frac{1}{m^{2}} \bigl\Vert \partial_{t} u^{\mathrm{TW}}_{t} \bigr\Vert ^{2} $$

and, as in (27),

$$\begin{array}{rl} S_{7,3} & \leq {∥\frac{F^{\prime \prime }(u_{t}^{\mathrm{TW}})}{F^{\prime }{\left(u_{t}^{\mathrm{TW}}\right)}^{2}}∥}_{\infty }^{2}{∥\left(\partial_{t}u_{t}^{\mathrm{TW}}-\pi^{m}\left(\partial_{t}u_{t}^{\mathrm{TW}}\right)\right)1_{\left(-L^{m},l^{m}\right)}∥}^{2}\\ & \leq \frac{1}{m^{2}}c^{2}{∥\frac{F^{\prime \prime }(u_{t}^{\mathrm{TW}})}{F^{\prime }{\left(u_{t}^{\mathrm{TW}}\right)}^{2}}∥}_{\infty }^{2}{∥{\hat{u}}_{xx}∥}^{2}. \end{array}$$

The last summand satisfies, using (23) and (27),

$$\begin{aligned} S_{7,4} \leq&3 \biggl\Vert \frac{F''(u^{\mathrm{TW}}_{t})}{F'(u^{\mathrm{TW}}_{t})^{2}} \biggr\Vert _{\infty}^{2} \\ &{}\times \biggl[ \sum_{k} \frac{1}{m} \biggl( \int_{-L^{m}}^{L^{m}} w\biggl(\frac {k}{m}-y\biggr) \bigl( F\bigl(u^{\mathrm{TW}}_{t}(y)\bigr)-\pi^{m}\bigl(F \bigl(u^{\mathrm{TW}}_{t}\bigr)\bigr) (y)\bigr)^{2} \,dy \biggr)^{2} \\ &{}+ S_{3} + S_{4} \biggr] \\ \leq&3 \biggl\Vert \frac{F''(u^{\mathrm{TW}}_{t})}{F'(u^{\mathrm{TW}}_{t})^{2}} \biggr\Vert _{\infty}^{2}\biggl[\biggl(1+ \frac{1}{m}\|w_{x}\|_{1}\biggr) \bigl\Vert F' \bigr\Vert _{\infty}^{2} \frac{1}{m^{2}} \|\hat {u}_{x}\|^{2} + S_{3} + S_{4}\biggr] . \end{aligned}$$

### A.2 The Itô Correction

We have

$$\begin{array}{rl} & {∥\sigma^{m}\left(t,v_{t}^{m}+\pi^{m}\left(u_{t}^{\mathrm{TW}}\right)\right)\circ \Phi^{m}-\sigma \left(t,v_{t}+u_{t}^{\mathrm{TW}}\right)∥}_{L_{2}^{0}}^{2}\\ & \;\leq 3({∥\left(\sigma^{m}\left(t,v_{t}^{m}+\pi^{m}\left(u_{t}^{\mathrm{TW}}\right)\right)-\sigma^{m}\left(t,\left(v_{t}+u_{t}^{\mathrm{TW}}\right)1_{\left(-L^{m},L^{m}\right)}\right)\right)\circ \Phi^{m}∥}_{L_{2}^{0}}^{2}\\ & \;+{∥\left(\sigma^{m}\left(t,\left(v_{t}+u_{t}^{\mathrm{TW}}\right)1_{\left(-L^{m},L^{m}\right)}\right)-\sigma \left(t,v_{t}+u_{t}^{\mathrm{TW}}\right)\right)\circ \Phi^{m}∥}_{L_{2}^{0}}^{2}\\ & \;+{∥\sigma \left(t,v_{t}+u_{t}^{\mathrm{TW}}\right)\circ \Phi^{m}-\sigma \left(t,v_{t}+u_{t}^{\mathrm{TW}}\right)∥}_{L_{2}^{0}}^{2})\\ & \;=:3(S_{8}+S_{9}+S_{10}). \end{array}$$

Let $(e_{k})$ be an orthonormal basis of $L^{2}(\mathbb {R})$. Note that by Parseval’s identity

$$\begin{array}{rl} & \sum_{k}{\left(\Phi^{m}(\sqrt{Q}e_{k})\right)}^{2}\\ & \;=\sum_{k}{\left(\sum_{l}2m\langle \sqrt{Q}e_{k},1_{J_{l}^{m}}\rangle 1_{I_{l}^{m}}\right)}^{2}\\ & \;=\sum_{l}\sum_{k}4m^{2}{\langle \sqrt{Q}1_{J_{l}^{m}},e_{k}\rangle }^{2}1_{I_{l}^{m}}=\sum_{l}4m^{2}{∥\sqrt{Q}1_{J_{l}^{m}}∥}^{2}1_{I_{l}^{m}}\\ & \;=\sum_{l}4m^{2}\int {\left(\int_{J_{l}^{m}}q(x,y)\;dy\right)}^{2}\;dx\;1_{I_{l}^{m}}\\ & \;\leq \sum_{l}2m\int \int_{J_{l}^{m}}q^{2}(x,y)\;dy\;dx\;1_{I_{l}^{m}}\leq \underset{x}{\sup}{∥q(x,\cdot )∥}^{2}1_{\left[-L^{m},L^{m}\right)}. \end{array}$$

Thus,

$$\begin{array}{rcl} S_{8} & = & \sum_{k}\int_{-\infty }^{\infty }(\sigma^{m}\left(t,v_{t}^{m}+\pi^{m}\left(u_{t}^{\mathrm{TW}}\right)\right)\\ & & -\sigma^{m}\left(t,\left(v_{t}+u_{t}^{\mathrm{TW}}\right)1_{\left(-L^{m},L^{m}\right)}\right){)}^{2}(x){\left(\Phi^{m}(\sqrt{Q}e_{k})\right)}^{2}(x)\;dx\\ & \leq & \underset{x}{\sup}{∥q(x,\cdot )∥}^{2}L_{\sigma }^{2}{∥v_{t}^{m}+\pi^{m}\left(u_{t}^{\mathrm{TW}}\right)-\left(v_{t}+u_{t}^{\mathrm{TW}}\right)1_{\left(-L^{m},L^{m}\right)}∥}^{2}\\ & \overset{\text{(27)}}{\leq } & 2\underset{x}{\sup}{∥q(x,\cdot )∥}^{2}L_{\sigma }^{2}\left({∥v_{t}^{m}-v_{t}∥}^{2}+\frac{1}{m^{2}}{∥{\hat{u}}_{x}∥}^{2}\right). \end{array}$$

Thus,

$$\begin{array}{rcl} S_{8} & = & \sum_{k}\int_{-\infty }^{\infty }(\sigma^{m}\left(t,v_{t}^{m}+\pi^{m}\left(u_{t}^{\mathrm{TW}}\right)\right)\\ & & -\sigma^{m}\left(t,\left(v_{t}+u_{t}^{\mathrm{TW}}\right)1_{\left(-L^{m},L^{m}\right)}\right){)}^{2}(x){\left(\Phi^{m}(\sqrt{Q}e_{k})\right)}^{2}(x)\;dx\\ & \leq & \underset{x}{\sup}{∥q(x,\cdot )∥}^{2}L_{\sigma }^{2}{∥v_{t}^{m}+\pi^{m}\left(u_{t}^{\mathrm{TW}}\right)-\left(v_{t}+u_{t}^{\mathrm{TW}}\right)1_{\left(-L^{m},L^{m}\right)}∥}^{2}\\ & \overset{\text{(27)}}{\leq } & 2\underset{x}{\sup}{∥q(x,\cdot )∥}^{2}L_{\sigma }^{2}\left({∥v_{t}^{m}-v_{t}∥}^{2}+\frac{1}{m^{2}}{∥{\hat{u}}_{x}∥}^{2}\right) \end{array}$$

and

$$\begin{array}{rl} S_{9} & ={∥\left(\sigma^{m}\left(t,\left(v_{t}+u_{t}^{\mathrm{TW}}\right)1_{\left(-L^{m},L^{m}\right)}\right)-\sigma \left(t,v_{t}+u_{t}^{\mathrm{TW}}\right)\right)\circ \Phi^{m}∥}_{L_{2}^{0}}^{2}\\ & \leq \underset{x}{\sup}{∥q(x,\cdot )∥}^{2}{∥\sigma^{m}\left(t,\left(v_{t}+u_{t}^{\mathrm{TW}}\right)1_{\left(-L^{m},L^{m}\right)}\right)-\sigma \left(t,v_{t}+u_{t}^{\mathrm{TW}}\right)∥}^{2}. \end{array}$$

Using Parseval’s identity again we get

$$\begin{array}{rcl} S_{10} & = & \int_{-\infty }^{\infty }\sigma {\left(t,v_{t}+u_{t}^{\mathrm{TW}}\right)}^{2}(x)\\ & & \times \sum_{k}{\left(\sum_{l}2m\langle \sqrt{Q}e_{k},1_{J_{l}^{m}}\rangle 1_{I_{l}^{m}}(x)-\sqrt{Q}e_{k}(x)\right)}^{2}\;dx\\ & = & \sum_{l}\int_{\frac{l}{m}}^{\frac{l+1}{m}}\sigma {\left(t,v_{t}+u_{t}^{\mathrm{TW}}\right)}^{2}(x)\\ & & \times \sum_{k}{\left(2m\int_{J_{l}^{m}}\int_{-\infty }^{\infty }\left(q(z,y)-q(x,y)\right)e_{k}(y)\;dy\;dz\right)}^{2}\;dx\\ & & +\left(\int_{-\infty }^{-L^{m}}+\int_{L^{m}}^{\infty }\right)\sigma {\left(t,v_{t}+u_{t}^{\mathrm{TW}}\right)}^{2}(x)\sum_{k}{\left(\sqrt{Q}e_{k}(x)\right)}^{2}\;dx\\ & \leq & \sum_{l}\int_{\frac{l}{m}}^{\frac{l+1}{m}}\sigma {\left(t,v_{t}+u_{t}^{\mathrm{TW}}\right)}^{2}(x){∥2m\int_{J_{l}^{m}}\left(q(z,\cdot )-q(x,\cdot )\right)\;dz∥}^{2}\;dx\\ & & +\left(\int_{-\infty }^{-L^{m}}+\int_{L^{m}}^{\infty }\right)\sigma {\left(t,v_{t}+u_{t}^{\mathrm{TW}}\right)}^{2}(x)\sum_{k}{\left(\int_{-\infty }^{\infty }q(x,y)e_{k}(y)\;dy\right)}^{2}\;dx\\ & \leq & {∥\sigma \left(t,v_{t}+u_{t}^{\mathrm{TW}}\right)∥}^{2}\underset{l}{\sup}\underset{x\in I_{l}^{m}}{\sup}{∥2m\int_{J_{l}^{m}}q(z,\cdot )\;dz-q(x,\cdot )∥}^{2}\\ & & +\underset{x}{\sup}{∥q(x,\cdot )∥}^{2}\left(\int_{-\infty }^{-L^{m}}+\int_{L^{m}}^{\infty }\right)\sigma {\left(t,v_{t}+u_{t}^{\mathrm{TW}}\right)}^{2}(x)\;dx. \end{array}$$

### A.3 Application of Gronwall’s Lemma

We use *K*, $K_{1}$, $K_{2}$, *K̃*, etc. to denote suitable constants that may differ from step to step. Summarizing the previous steps and using Young’s inequality we arrive at

$$\begin{aligned} & \frac{1}{2} d \bigl\Vert v^{m}_{t}-v_{t} \bigr\Vert ^{2} \\ &\quad \leq \biggl[- \bigl\Vert v^{m}_{t}-v_{t} \bigr\Vert ^{2} + \frac{1}{2} \bigl\Vert v^{m}_{t}-v_{t} \bigr\Vert ^{2} \\ & \qquad {} + \frac{1}{2} \bigl\Vert v^{m}_{t} - v_{t} + b^{m}\bigl(t, v^{m}_{t}+ \pi^{m}\bigl(u^{\mathrm{TW}} _{t}\bigr)\bigr) - b\bigl(t, v_{t}+u^{\mathrm{TW}}_{t}\bigr) \\ &\qquad {}- \pi^{m}\bigl( \partial_{t}u^{\mathrm{TW}}_{t}\bigr) + \partial_{t} u^{\mathrm{TW}}_{t} \bigr\Vert _{2}^{2}+ \frac{1}{4N} \bigl\Vert v^{m}_{t}-v_{t} \bigr\Vert ^{2} \\ &\qquad {} + \frac{1}{4N} \biggl\Vert \frac {F''(u^{\mathrm{TW}}_{t})}{F'(u^{\mathrm{TW}}_{t})^{2}} \partial_{t} u^{\mathrm{TW}}_{t} - \pi^{m} \biggl( \frac{F''(u^{\mathrm{TW}} _{t})}{F'(u^{\mathrm{TW}}_{t})^{2}}\biggr) b^{m}\bigl(t,\pi^{m} \bigl(u^{\mathrm{TW}}_{t}\bigr)\bigr) \biggr\Vert ^{2} \biggr] \,dt \\ &\qquad {} + \frac{1}{2} \bigl\Vert \sigma^{m} \bigl(t,v^{m}_{t}+ \pi^{m}\bigl(u^{\mathrm{TW}}_{t} \bigr)\bigr) \circ\varPhi ^{m} - \sigma\bigl(t,v_{t}+ u^{\mathrm{TW}}_{t}\bigr) \bigr\Vert _{L^{0}_{2}}^{2} \,dt \\ &\qquad {} + \bigl\langle v^{m}_{t}-v_{t}, \bigl( \sigma^{m}\bigl(t, v^{m}_{t}+\pi^{m} \bigl(u^{\mathrm{TW}} _{t}\bigr)\bigr) \circ\varPhi^{m} - \sigma\bigl(t, v_{t}+u^{\mathrm{TW}}_{t}\bigr) \bigr) \,d \mathcal{W}^{Q}_{t} \bigr\rangle \\ &\quad \leq K_{1} \bigl\Vert v_{t}^{m}-v_{t} \bigr\Vert ^{2} \,dt + K_{2} R(t,v_{t},m) \,dt + dM_{t}, \end{aligned}$$

where

$$\begin{array}{rcl} R(t,v_{t},m) & = & \frac{1}{m^{2}}\left({∥v_{t}∥}^{2}+{∥{\hat{u}}_{x}∥}^{2}+{∥{\hat{u}}_{xx}∥}^{2}\right)\\ & & +\left(\int_{L^{m}}^{\infty }+\int_{-\infty }^{-L^{m}}\right)(v_{t}^{2}(x)+{\left(w*|v_{t}|\right)}^{2}(x)\\ & & +\sigma {\left(t,v_{t}+u_{t}^{\mathrm{TW}}\right)}^{2}(x)+{\left(\partial_{x}u_{t}^{\mathrm{TW}}(x)\right)}^{2})\;dx\\ & & +{∥\sigma \left(t,v_{t}+u_{t}^{\mathrm{TW}}\right)∥}^{2}\underset{k}{\sup}\underset{x\in I_{k}^{m}}{\sup}{∥2m\sqrt{Q}1_{J_{k}^{m}}-q(x,\cdot )∥}^{2}\\ & & +{∥\sigma^{m}\left(t,\left(v_{t}+u_{t}^{\mathrm{TW}}\right)1_{\left(-L^{m},L^{m}\right)}\right)-\sigma \left(t,v_{t}+u_{t}^{\mathrm{TW}}\right)∥}^{2} \end{array}$$

and

$$M_{t} = \int_{0}^{t} \bigl\langle v^{m}_{s}-v_{s}, \bigl(\sigma^{m}\bigl(s, v^{m}_{s}+ \pi^{m}\bigl(u^{\mathrm{TW}}_{s}\bigr)\bigr) \circ \varPhi^{m} - \sigma\bigl(s, v_{s}+u^{\mathrm{TW}}_{s} \bigr) \bigr) \,d\mathcal{W}^{Q}_{s} \bigr\rangle $$

is a martingale with quadratic variation process

28 $$\begin{aligned}{} [M ]_{t} = & \int_{0}^{t} \sum_{k} \bigl\langle v^{m}_{s}-v_{s}, \bigl( \sigma^{m}\bigl(s, v^{m}_{s}+\pi^{m} \bigl(u^{\mathrm{TW}} _{s}\bigr)\bigr) \circ\varPhi^{m} \\ & {} - \sigma\bigl(s, v_{s}+u^{\mathrm{TW}}_{s}\bigr) \bigr) \circ\sqrt{Q} e_{k} \bigr\rangle ^{2} \,ds \\ \leq& \int_{0}^{t} \bigl\Vert v^{m}_{s} - v_{s} \bigr\Vert ^{2} \bigl\Vert \bigl( \sigma^{m}\bigl(s, v^{m}_{s}+\pi ^{m} \bigl(u^{\mathrm{TW}}_{s}\bigr)\bigr) \circ\varPhi^{m} \\ & {} - \sigma\bigl(s, v_{s}+u^{\mathrm{TW}}_{s}\bigr) \bigr) \bigr\Vert _{L_{2}^{0}}^{2} \,ds. \end{aligned}$$

Applying Itô’s formula to the real-valued stochastic process $\| v^{m}_{t}-v_{t}\|^{2}$ we obtain for $p\geq2$

$$\begin{aligned} & d \bigl\Vert v^{m}_{t}-v_{t} \bigr\Vert ^{p} \\ &\quad = \frac{p}{2} \bigl\Vert v^{m}_{t}-v_{t} \bigr\Vert ^{p-2} d \bigl\Vert v^{m}_{t}-v_{t} \bigr\Vert ^{2} \\ &\qquad {}+ \frac{p (p-2)}{8} \bigl\Vert v^{m}_{t}-v_{t} \bigr\Vert ^{p-4} d \bigl[ \bigl\Vert v^{m}_{t}-v_{t} \bigr\Vert ^{2} \bigr]_{t} \\ &\quad \stackrel{\text{(28)}}{\leq} K_{1} p \bigl\Vert v^{m}_{t}-v_{t} \bigr\Vert ^{p} \,dt + K_{2} p \bigl\Vert v^{m}_{t}-v_{t} \bigr\Vert ^{p-2} R(t,v_{t},m) \,dt \\ &\qquad {}+ p \bigl\Vert v^{m}_{t}-v_{t} \bigr\Vert ^{p-2} \,dM_{t}+ \frac{p(p-2)}{2} \bigl\Vert v^{m}_{t}-v_{t} \bigr\Vert ^{p-2} \\ &\qquad {}\times \bigl\Vert \bigl( \sigma ^{m}\bigl(t, v^{m}_{t}+\pi^{m}\bigl(u^{\mathrm{TW}}_{t} \bigr)\bigr) \circ\varPhi^{m} - \sigma\bigl(t, v_{t}+u^{\mathrm{TW}}_{t} \bigr) \bigr) \bigr\Vert _{L_{2}^{0}}^{2} \,dt. \end{aligned}$$

Estimating the last term as above and using Young’s inequality we obtain

$$\begin{aligned} & d \bigl\Vert v^{m}_{t}-v_{t} \bigr\Vert ^{p} \\ &\quad \leq\tilde{K_{1}} \bigl\Vert v^{m}_{t}-v_{t} \bigr\Vert ^{p} \,dt + \tilde{K_{2}} \bigl\Vert v^{m}_{t}-v_{t} \bigr\Vert ^{p-2} R(t,v_{t},m) \,dt \\ &\qquad {}+ p \bigl\Vert v^{m}_{t}-v_{t} \bigr\Vert ^{p-2} \,dM_{t} \\ &\quad \leq \biggl(\tilde{K_{1}}+\tilde{K_{2}} \frac{p-2}{p} \biggr) \bigl\Vert v^{m}_{t}-v_{t} \bigr\Vert ^{p} \,dt + \tilde{K_{2}}\frac{2}{p} R(t,v_{t},m)^{\frac{p}{2}} \,dt \\ &\qquad {}+ p \bigl\Vert v^{m}_{t}-v_{t} \bigr\Vert ^{p-2} \,dM_{t}. \end{aligned}$$

Integrating, maximizing over $t\leq T$, and taking expectations we get

29 $$\begin{aligned} & E \Bigl( \sup_{t\leq T} \bigl\Vert v^{m}_{t}-v_{t} \bigr\Vert ^{p} \Bigr) \\ &\quad \leq \bigl\Vert v^{m}_{0}-v_{0} \bigr\Vert ^{p} + \biggl(\tilde{K_{1}}+\tilde{K_{2}} \frac {p-2}{p} \biggr) E \biggl( \sup_{t\leq T} \int_{0}^{t} \bigl\Vert v^{m}_{s}-v_{s} \bigr\Vert ^{p} \,ds \biggr) \\ &\qquad {} + \tilde{K_{2}}\frac{2}{p} E \biggl(\sup _{t\leq T} \int_{0}^{t} R(s,v_{s},m)^{\frac{p}{2}} \,ds \biggr) + p E \biggl(\sup_{t\leq T} \int_{0}^{t} \bigl\Vert v^{m}_{s}-v_{s} \bigr\Vert ^{p-2} \,dM_{s} \biggr) \\ &\quad \leq \bigl\Vert v^{m}_{0}-v_{0} \bigr\Vert ^{p} + \biggl(\tilde{K_{1}}+\tilde{K_{2}} \frac {p-2}{p} \biggr) \int_{0}^{T} E \Bigl( \sup_{s\leq t} \bigl\Vert v^{m}_{s}-v_{s} \bigr\Vert ^{p} \Bigr) \,dt \\ &\qquad {} + \tilde{K_{2}}\frac{2}{p} E \biggl( \int_{0}^{T} R(s,v_{s},m)^{\frac{p}{2}} \,ds \biggr) + p E \biggl(\sup_{t\leq T} \int_{0}^{t} \bigl\Vert v^{m}_{s}-v_{s} \bigr\Vert ^{p-2} \,dM_{s} \biggr). \end{aligned}$$

We estimate the last term using the Burkholder–Davis–Gundy inequality, (28), and Young’s inequality:

$$\begin{aligned} & p E \biggl(\sup_{t\leq T} \int_{0}^{t} \bigl\Vert v^{m}_{s}-v_{s} \bigr\Vert ^{p-2} \,dM_{s} \biggr) \\ & \quad \leq K E \biggl( \int_{0}^{T} \bigl\Vert v_{s}^{m}-v_{s} \bigr\Vert _{2}^{2p-2} \\ &\qquad {}\times\bigl\Vert \bigl( \sigma^{m}\bigl(s, v^{m}_{s}+\pi^{m} \bigl(u^{\mathrm{TW}}_{s}\bigr)\bigr) \circ\varPhi^{m} - \sigma\bigl(s, v_{s}+u^{\mathrm{TW}}_{s}\bigr) \bigr) \bigr\Vert _{L_{2}^{0}}^{2} \,ds \biggr)^{\frac{1}{2}} \\ &\quad \leq K E \biggl( \sup_{t\leq T} \bigl\Vert v_{t}^{m}-v_{t} \bigr\Vert ^{p-1} \\ &\qquad {}\times \biggl( \int_{0}^{T} \bigl\Vert \bigl( \sigma^{m}\bigl(s, v^{m}_{s} + \pi^{m} \bigl(u^{\mathrm{TW}}_{s}\bigr)\bigr) \circ\varPhi^{m} - \sigma\bigl(s, v_{s} + u^{\mathrm{TW}}_{s}\bigr) \bigr) \bigr\Vert _{L_{2}^{0}}^{2} \,ds \biggr)^{\frac{1}{2}} \biggr) \\ &\quad \leq\frac{1}{2} E \Bigl( \sup_{t\leq T} \bigl\Vert v_{t}^{m}-v_{t} \bigr\Vert ^{p} \Bigr) \\ &\qquad {} + \tilde{K}_{3} E \biggl( \int_{0}^{T} \bigl\Vert \bigl( \sigma^{m}\bigl(s, v^{m}_{s} +\pi^{m} \bigl(u^{\mathrm{TW}}_{s}\bigr)\bigr) \circ\varPhi^{m} - \sigma\bigl(s, v_{s}+u^{\mathrm{TW}}_{s}\bigr) \bigr) \bigr\Vert _{L_{2}^{0}}^{2} \,ds \biggr)^{\frac{p}{2}}. \end{aligned}$$

Bringing the first summand to the left-hand side of (29) this implies that

$$\begin{aligned} & E \Bigl( \sup_{t\leq T} \bigl\Vert v^{m}_{t}-v_{t} \bigr\Vert ^{p} \Bigr) \\ &\quad \leq2 \bigl\Vert v^{m}_{0}-v_{0} \bigr\Vert ^{p} + 2 \biggl(\tilde{K_{1}}+\tilde{K_{2}} \frac {p-2}{p} \biggr) \int_{0}^{T} E \Bigl( \sup_{s\leq t} \bigl\Vert v^{m}_{s}-v_{s} \bigr\Vert ^{p} \Bigr)\,dt \\ &\qquad {} + \tilde{K_{2}}\frac{4}{p} E \biggl( \int_{0}^{T} R(s,v_{s},m)^{\frac{p}{2}} \,ds \biggr) \\ &\qquad {}+ 2 \tilde{K_{3}} E \biggl( \int_{0}^{T} \bigl\Vert \bigl( \sigma^{m}\bigl(t, v^{m}_{t}+\pi^{m} \bigl(u^{\mathrm{TW}}_{t}\bigr)\bigr) \circ\varPhi^{m} - \sigma\bigl(t, v_{t}+u^{\mathrm{TW}}_{t}\bigr) \bigr) \bigr\Vert _{L_{2}^{0}}^{2} \,ds \biggr)^{\frac{p}{2}}. \end{aligned}$$

We estimate the last term as before and obtain

$$\begin{aligned} & E \biggl( \int_{0}^{T} \bigl\Vert \bigl( \sigma^{m}\bigl(s, v^{m}_{s} + \pi^{m} \bigl(u^{\mathrm{TW}}_{s}\bigr)\bigr) \circ \varPhi^{m} - \sigma\bigl(s, v_{s}+u^{\mathrm{TW}}_{s}\bigr) \bigr) \bigr\Vert _{L_{2}^{0}}^{2} \,ds \biggr)^{\frac {p}{2}} \\ &\quad \leq K E \biggl( \int_{0}^{T} \bigl\Vert v^{m}_{s}-v_{s} \bigr\Vert ^{2} + R(s, v_{s},m) \,ds \biggr)^{\frac{p}{2}} \\ &\quad \leq\tilde{K} E \biggl( \int_{0}^{T} \sup_{s\leq t} \bigl\Vert v^{m}_{s}-v_{s} \bigr\Vert ^{p} \,dt + \int_{0}^{T} R(s, v_{s},m)^{\frac{p}{2}} \,ds \biggr). \end{aligned}$$

Altogether we arrive at

$$\begin{aligned} E \Bigl( \sup_{t\leq T} \bigl\Vert v^{m}_{t}-v_{t} \bigr\Vert ^{p} \Bigr) \leq&2 \bigl\Vert v^{m}_{0} -v_{0} \bigr\Vert _{2}^{p} + \hat{K_{1}} E \int_{0}^{T} R(t,v_{t},m)^{\frac{p}{2}} \,dt \\ &{} {} + \hat{K_{2}} \int_{0}^{T} E \Bigl( \sup_{s\leq t} \bigl\Vert v^{m}_{s}-v_{s} \bigr\Vert ^{p} \Bigr) \,dt. \end{aligned}$$

An application of Gronwall’s lemma yields

$$ E \Bigl( \sup_{t\leq T} \bigl\Vert v^{m}_{t}-v_{t} \bigr\Vert ^{p} \Bigr) \leq K \Bigl( \bigl\Vert v^{m}_{0}-v_{0} \bigr\Vert ^{p} + E \Bigl( \sup_{t\leq T} R(t,v_{t},m)^{\frac{p}{2}} \Bigr) \Bigr). $$

The sequence of continuous functions $f^{m}: [0,T] \rightarrow \mathbb {R}$

$$\begin{aligned} \begin{aligned} f^{m}(t) ={}& \biggl( \biggl( \int_{L^{m}}^{\infty} + \int_{-\infty}^{-L^{m}} \biggr) v_{t}^{2}(x) + \bigl(w\ast \vert v_{t} \vert \bigr)^{2}(x) \\ &{}+ \sigma \bigl(t,v_{t}+u^{\mathrm{TW}}_{t}\bigr)^{2}(x) + \bigl(\partial_{x} u^{\mathrm{TW}}_{t}(x) \bigr)^{2} \,dx \biggr)^{\frac{p}{2}} \end{aligned} \end{aligned}$$

is decreasing and converges pointwise to 0 since all the integrands are in $L^{2}(\mathbb {R})$. By Dini’s theorem the convergence is uniform. This together with the facts that ${\|\sigma(t,v_{t})\|_{2}^{2} \leq K(1+\|v_{t}\| ^{2})}$ and ${E ( \sup_{t\leq T} \|v_{t}\|^{2} ) < \infty}$ by Proposition 3, assumptions (i) and (ii), and dominated convergence implies that

$$\begin{array}{rl} & E\left(\underset{t\leq T}{\sup}R{\left(t,v_{t},m\right)}^{\frac{p}{2}}\right)\\ & \;\leq K(\frac{1}{m^{p}}\left(E\left(\underset{t\leq T}{\sup}{∥v_{t}∥}^{p}\right)+{∥{\hat{u}}_{x}∥}^{p}+{∥{\hat{u}}_{xx}∥}^{p}\right)+E\left(\underset{t\leq T}{\sup}f^{m}(t)\right)\\ & \;+E\left(\underset{t\leq T}{\sup}{∥\sigma^{m}\left(t,\left(v_{t}+u_{t}^{\mathrm{TW}}\right)1_{\left(-L^{m},L^{m}\right)}\right)\circ \Phi^{m}-\sigma \left(t,v_{t}+u_{t}^{\mathrm{TW}}\right)∥}^{p}\right)\\ & \;+E\left(\underset{t\leq T}{\sup}{∥\sigma \left(t,v_{t}+u_{t}^{\mathrm{TW}}\right)∥}^{p}\right)\underset{k}{\sup}\underset{x\in I_{k}^{m}}{\sup}{∥2m\sqrt{Q}1_{J_{k}^{m}}-q(x,\cdot )∥}^{p})\overset{m\to \infty }{\to }0 \end{array}$$

and hence

$$E \Bigl( \sup_{t\leq T} \bigl\Vert v^{m}_{t}-v_{t} \bigr\Vert ^{p} \Bigr) \xrightarrow{m\rightarrow \infty}0. $$
